# Semiquantitative 2-[^18^F]FDG PET/CT-based parameters role in lymphoma

**DOI:** 10.3389/fmed.2024.1515040

**Published:** 2024-12-18

**Authors:** Domenico Albano, Marco Ravanelli, Rexhep Durmo, Annibale Versari, Angelina Filice, Alessio Rizzo, Manuela Racca, Daniele Antonio Pizzuto, Francesco Bertagna, Salvatore Annunziata

**Affiliations:** ^1^Nuclear Medicine Unit, ASST Spedali Civili of Brescia, Brescia, Italy; ^2^Department of Nuclear Medicine, University of Brescia, Brescia, Italy; ^3^Radiology Unit, University of Brescia, Brescia, Italy; ^4^Nuclear Medicine Unit, Azienda Unità Sanitaria Locale-IRCCS di Reggio Emilia, Reggio Emilia, Italy; ^5^Division of Nuclear Medicine, Candiolo Cancer Institute, FPO-IRCCS, Turin, Italy; ^6^Dipartimento di Radiodiagnostica e Radioterapia, Fondazione Policlinico Universitario A. Gemelli IRCCS, Rome, Italy

**Keywords:** PET/CT, lymphoma, FDG, sarcopenia, MTV, TLG, Dmax, radiomics

## Abstract

2-deoxy-2-[^18^F]-fluoro-D-glucose (2-[^18^F]FDG) positron emission tomography/computed tomography (PET/CT) plays a crucial role in the management of lymphoma in different settings, such as staging disease, assessing response to therapy, predicting prognosis, and planning RT. Beside visual analysis, several semiquantitative parameters were introduced to study lymphoma with promising results. These parameters can represent different disease characteristics, like body composition (such as sarcopenic index), dissemination of disease (Dmax), tumor burden (including metabolic tumor volume) and texture features. All these parameters showed promising results, especially in terms of prognosis (progression free survival and overall survival), but lack of standardization and shared methodology remains a big issue. Advances in PET-based biomarkers are on the horizon, yet their integration into clinical decision-making is currently hindered by methodological limitations that require resolution through confirmatory prospective validation in specific patient groups. This review highlights studies demonstrating the prognostic and predictive value of these semiquantitative parameters in lymphoma, while also discussing their potential applicability in clinical practice.

## 1 Introduction

Lymphoma is a heterogeneous group of tumors of the hematopoietic and lymphoid tissue, divided in two main categories: Hodgkin Lymphoma (HL) and non-Hodgkin Lymphoma (NHL). NHL consist of 90% of all lymphoma. Lymphoma represents approximately 5% of all malignancies. Lymphoma can be aggressive or indolent according to the main histological features and treatments are directly related to their aggressiveness. 2-deoxy-2-[^18^F]-fluoro-D-glucose (2-[^18^F]FDG) positron emission tomography/computed tomography (PET/CT) is a hybrid imaging tool that has recently experienced a wide increase in its use and applications. 2-[^18^F]FDG exploits the capacity to detect lesions with high activity based on their increased glycolytic metabolism. The role of 2-[^18^F]FDG PET/CT in lymphoma is well established with strong evidence in staging disease and evaluation of treatment response in FDG-avid lymphoma, which for definition are considered HL, Follicular Lymphoma (FL) and Diffuse Large B cell Lymphoma (DLBCL) (1, 2). Recent data described the potential usefulness of 2-[^18^F]FDG PET/CT also in other less studied lymphoma variants, like Mantle cell lymphoma (MCL) (3), Burkitt Lymphoma (BL) (4) and Marginal Lymphoma (5). Moreover, a prognostic impact of PET/CT features was demonstrated both for progression-free survival (PFS) and overall survival (OS) (6). In 2014, a multidisciplinary panel of lymphoma experts established the Lugano criteria, which include the previously developed 5-point scale (5-PS), known as the Deauville score. This scale relies on the visual evaluation of FDG uptake in the reference lesion, compared to reference organs such as the liver and mediastinum, to assess treatment response (7, 8). This visual score quickly acquired universal acceptance and was commonly incorporated for response assessment in clinical practice and as a surrogate endpoint in clinical trials. However, more recently some semiquantitative parameters derived from PET or CT images were studied with promising findings, especially in the prognostic field. These biomarkers are derived from PET/CT scans, which are processed using molecular imaging algorithms and then transformed into quantitative variables (9). These variables encompass various disease characteristics, including volume, represented as metabolic tumor volume (MTV), which is typically defined as the hypermetabolic tumor burden; features of dissemination, such as Dmax, which measures the distance between areas of increased uptake; and skeletal muscle status, often described in terms of sarcopenic features or image texture. Despite extensive development efforts, these semiquantitative PET-based biomarkers remain largely excluded from risk-adapted treatment approaches and are limited only in the research field. The aim of this narrative review is to resume the most relevant applications and findings of these PET-metrics in lymphoma, underlying their strengths and limitations, as well as recent efforts to implement PET/CT-based metrics as promising tools for precision medicine.

## 2 Sarcopenia

### 2.1 Definition and background

The term sarcopenia was first coined by Irwin Rosenberg in 1988 with this definition: *a syndrome characterized by progressive and generalized loss of skeletal muscle mass (SMM) and strength, associated with adverse outcomes like physical disability, poor quality of life and death* (10). Although primarily associated with aging, sarcopenia is also prevalent in pathological conditions like inflammatory diseases, endocrine disorders, chronic illnesses and nutritional deficiencies (11). In 2019, the European Working Group on Sarcopenia in Older People (EWGSOP2) established 3 diagnostic criteria: decreased muscle quality or quantity, decreased muscle strength and decreased physical performance (12). Skeletal muscle (SM), which accounts approximately for half of total body mass plays several crucial functions, like locomotion and homeostase. SM cells product many specific cytokines, named myokines, which have paramount paracrine and endocrine activities (13, 14). Besides, some of these cytokines may have antineoplastic effects. Consequently, sarcopenia is considered a poor prognostic marker in many oncological diseases. It is associated with poor prognosis, expressed as PFS or OS, and with increased drug-related toxicities (15). There is substantial evidence supporting the role of sarcopenia in predicting prognosis in hematologic malignancies, particularly in lymphoma (16–19). Therefore, it is essential to assess sarcopenia using precise and reproducible methods.

### 2.2 Technical characteristics

Over time, various imaging techniques have been explored for diagnosing sarcopenia, each with distinct characteristics regarding availability, cost, and ease of implementation. These include the grip test, which uses a calibrated hand-held dynamometer to assess muscle strength; the short physical performance battery to evaluate physical function; and computed tomography (CT)/magnetic resonance imaging (MRI), which provide quantitative estimates of muscle and fat tissue areas (12). CT and MRI are considered the best tools for these measurements due to their ability to differentiate fat from other soft tissues of the body, like muscle.

First, Shen et al. (20) reported that a single cross-sectional image at the level of the third lumbar vertebral accurately represents total body muscle mass. Then, Mourtzakis et al. (21) validated this method among oncological patients using CT images as a reference. Since then, many authors studied the clinical impact of decreased skeletal muscle mass as the representation of sarcopenia state.

### 2.3 Main results in lymphoma

**Figure 1:**
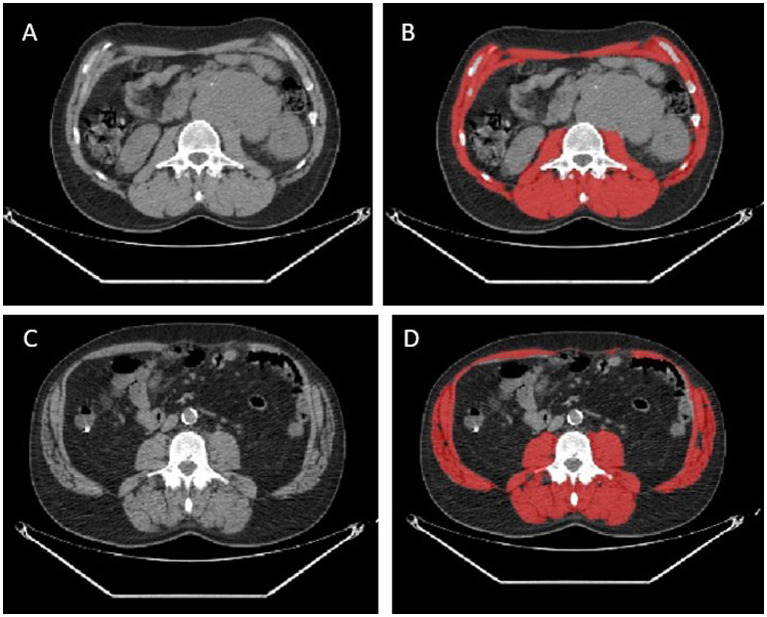
Two representative cases of patients with no sarcopenia detected by CT images **(A,B)** and sarcopenia **(C,D)**. In red the measurement of skeletal muscle area by the software.

A total of 38 studies accounting for 6,006 patients analyzed the role of sarcopenia measured by CT in lymphoma (22–59) (Table 1). The most common lymphoma histotype studied was DLBCL, followed by HL and MCL. The rate of sarcopenia reported was very heterogeneous ranging from 16 to 73% and dependent to the heterogeneous nature of the population analyzed. Now, CT is considered the ideal tool for the measurements of sarcopenia and muscle measurement at the level of the third lumbar vertebra (L3) the most frequent site for this kind of analysis. The procedure consists of the use of cross-sectional area to measure psoas muscle and/or paravertebral lumbar muscles with specific software (Figure 1). For estimating sarcopenia, in most cases “high-dose” CT was chosen as imaging technique (22–33, 35, 37, 38, 40–44, 50–55, 58, 59), while in more recent studies a combination of high-dose CT and low- dose CT of PET was utilized (34, 36, 39, 46, 56), and in other only low- dose CT of PET (45, 47–49, 57). The CT component of hybrid PET/CT is utilized to correct attenuation in PET emission data and to ensure precise anatomical localization of radiotracer uptake seen in the PET images. In the context of lymphoma, the potential to apply PET/CT for sarcopenic assessment may streamline the process, as FDG PET/CT offers enhanced diagnostic accuracy in staging both nodal and extranodal disease when compared to CT alone. In comparative research high-dose CT and the CT component of PET/CT demonstrated to be accurate and reproducible in calculating the extent of skeletal muscle mass and adipose tissue (60). In addition to L3, some authors measured skeletal muscle mass in different anatomical sites, like the fourth thoracic vertebra (T4) (37), pectoralis muscle (31, 35) or the proximal thigh (45). The most commonly assessed parameter representing sarcopenia was the skeletal muscle index (SMI) expressed as cm^2^/m^2^, which usually represents the sum of areas of skeletal muscles in an axial slice region normalized for height. The muscles present at the L3 are psoas, abdominal transverse rectum, paraspinal, external, and internal oblique muscles. Some authors measured (24, 42, 44) only psoas areas and the subsequent parameter extracted was defined as the psoas muscle index (PMI). One of the open issues present in the literature is the presence of different thresholds of SMI for the definition of sarcopenia and these cut-offs are dependent on gender and body mass index (36, 39, 45, 52). As shown in Table 2, the most commonly used threshold for L3 SMI was 55.8 cm^2^/m^2^ for males and 38.9 cm^2^/m^2^ for females. However, the suggested threshold values varied widely, depending on the population studied, ranging from 43 cm^2^/m^2^ to 56.8 cm^2^/m^2^ for men and from 31 cm^2^/m^2^ to 47.4 cm^2^/m^2^ for women. For pectoralis muscle SMI, the typical cut-off values are 4.4 cm^2^/m^2^ for men and 3.1 cm^2^/m^2^ for women, while for PMI, they are 4.4 cm^2^/m^2^ for men and 3.1 cm^2^/m^2^ for women, or 6.36 cm^2^/m^2^ for men and 3.92 cm^2^/m^2^ for women. In general, sarcopenia metrics showed correlations with OS (22, 28, 30–32, 34–36, 43, 44, 46, 47, 51, 52, 54, 55, 57, 59) and PFS (23, 28, 30–32, 34–36, 41, 44, 46–48, 51, 52, 54, 57, 59) but in some studies (25, 29, 38, 39, 45, 49, 50, 53) this correlation was not revealed. Concerning treatment response evaluation, only articles investigating DLBCL receiving R-CHOP chemotherapy are present (28, 31, 35, 44, 58) and show a significant relationship with treatment response. Regarding the side effects and toxicities after therapies, seven publications (24, 26, 28, 31, 33, 39, 43) showed a significant association between sarcopenia and these complications.

**Table 1:** The main technical and clinical features.

First author (ref)	Year	Country	Study design	N° patients included	Female (%)	Mean/median (range)	Lymphoma histotype	Treatment	N° patients with sarcopenia (%)
Camus V (22)	2014	France	R	80	45 (56%)	78.66 (68–93)	DLBCL	R-CHOP (*n* = 44) R-miniCHOP (*n* = 36)	44 (55%)
Lanic H (23)	2014	France	R	82	46 (56%)	78 (68–93)	DLBCL	R-CHOP 8 (*n* = 45) R-miniCHOP (*n* = 37)	45 (55%)
Caram MV (24)	2015	United States of America	P	121	48 (40%)	53 (21–74)	DLBCL (*n* = 53) MCL (*n* = 33) HL (*n* = 17) FL (*n* = 5) other (*n* = 13)	HSCT	na
Nakamura N (25)	2015	Japan	R	207	86 (42%)	67 (19–86)	DLBCL	R-CHOP (*n* = 116) R-THP-COP (*n* = 91)	115 (56%)
Xiao DY (26)	2016	United States of America	R	522	12 (2%)	64	DLBCL	CHOP +/− R	245 (47%)
Xiao DY (27)	2016	United States of America	R	342	11 (3%)	63.4	DLBCL	CHOP +/− R	na
Go S (28)	2016	Korea	R	187	75 (40%)	(17–89)	DLBCL	R-CHOP	46 (24%)
Karmali R (29)	2017	United States of America	R	86	46 (53%)	64	DLBCL (*n* = 76) MCL (*n* = 10)	R-CHOP (*n* = 67) DA-EPOCH (*n* = 7) mixed (*n* = 12)	43 (50%)
Chu MP (30)	2017	Canada	R	224	99 (49%)	62(21–88)	DLBCL	R-CHOP	116 (52%)
Go S (31)	2017	Korea	R	193	81 (42%)	(21–86)	DLBCL	R-CHOP	77 (40%)
Jabbour J (32)	2018	Lebanon	R	93	41 (44%)	38 (17–70)	HL (*n* = 45) NHL (mixed T and B cell) (*n* = 48)	HSCT	na
DeFilipp Z (33)	2018	United States of America	R	315	127 (41%)	55 (19–77)	NHL B cell (*n* = 224) NHL T cell(*n* = 64) HL (*n* = 27)	HSCT	155 (49%)
Burkart M (34)	2019	United States of America	R	109	56 (51%)	nr	DLBCL (*n* = 89) MCL (*n* = 18) BL (*n* = 2)	chemotherapy	65 (60%)
Go S (35)	2020	Korea	R	228	98 (43%)	(21–88)	DLBCL	R-CHOP	100 (45%)
Lin RJ (36)	2020	United States of America	R	146	44 (30%)	61 (50–79)	NHL (*n* = 138) HL (*n* = 8)	HSCT	80 (55%)
Mishra S (37)	2020	United States of America	R	296	135 (46%)	52.4	NHL (*n* = 165) HL (*n* = 14) other (*n* = 117)	HSCT	182 (61%)
Rier HN (38)	2020	Netherlands	R	164	84 (52%)	64.5	DLBCL	R-CHOP	80 (49%)
Armenian SH (39)	2020	United States of America	R	320	122 (38%)	53.3 (18.5–78.1)	DLBCL (*n* = 133) HL (*n* = 84) MCL (*n* = 50) FL (*n* = 24) T-cell L (*n* = 21) other (*n* = 8)	HSCT	84 (26%)
Bas V (40)	2021	Turkey	R	59	25(42%)	39.5 (20–73)	HL	ABVD	na
Lucjanic M (41)	2021	Croatia	R	49	24(49%)	36	HL	ABVD (*n* = 38) eBEACOP (*n* = 11)	na
Hirota K (42)	2021	Japan	R	40	16 (40%)	58 (80–74)	malignant lymphoma	HSCT	na
Guo J (43)	2021	China	R	201	87 (43%)	56.9	DLBCL	R-CHOP	na
Iltar U (44)	2021	Turkey	R	120	54 (45%)	56.1 (52–68)	DLBCL	R-CHOP	65 (54%)
Besutti G (45)	2021	Italy	R	116	56 (48%)	63.7	DLBCL	R-CHOP (*n* = 70) R-mini CHOP (*n* = 18) R-MACOP-B (*n* = 9) R-CVP (*n* = 5) R-CODOX-M/R-IVAC (*n* = 3) EPOCH-R (*n* = 3)	29 (25%)
Zilioli V (46)	2021	Italy	R	154	76 (49%)	71	HL	ABVD (*n* = 117) mixed (*n* = 31) RT alone (*n* = 5)	113 (73%)
Albano D (47)	2022	Italy	R	88	47 (53%)	72.8 (65–91)	HL	ABVD (*n* = 63) mixed (*n* = 25)	58 (66%)
Albano D (48)	2022	Italy	R	53	14 (27%)	72.7 (66–88)	MCL	R-BAC (*n* = 22) R-CHOP (*n* = 10) other (*n* = 21)	32 (60%)
Tan X (49)	2022	China	R	14	35 (29%)	26 (3–64, 161, 162)	T-LBL	chemotherapy + intratecal therapy (*n* = 31) chemotherapy + HSCT (*n* = 12) chemotherapy + RT (*n* = 2)	18 (37%)
Penichoux J (50)	2023	France	P	95	48 (51%)	78.4 (70–92)	DLBCL	R-CHOP (*n* = 54) R-miniCHOP (*n* = 40)	53 (56%)
Go S-I (51)	2023	Korea	R	305	180 (59%)	66.5 (50–75)	DLBCL	R-CHOP	91 (42%)
Liao PH (52)	2023	Taiwan	R	67	30 (45%)	77.4 (70–91)	DLBCL	R-CHOP	
Aleixo GFP (53)	2023	United States of America	R	264	91 (34%)	59 (21–78)	NHL	HSCT	124 (47%)
Chen Y (54)	2023	China	R	181	82 (45%)	60 (22–83)	DLBCL	R-CHOP	75 (41%)
Rejeski K (55)	2023	Germany	R	106	40 (36%)	64 (19–83)	DLBCL	Car-T cell therapy	na
Sumransub N (56)	2023	United States of America	R	78	27 (35%)	58.9 (16.8–72)	DLBCL (*n* = 30) MCL (*n* = 26) HL (*n* = 13) Other(*n* = 9)	HSCT	27 (35%)
Tan X (57)	2024	China	R	103	53 (52%)	54 (21–76)	DLBCL	R-CHOP	30 (29%)
Surov A (58)	2024	Germany	R	61	29 (48%)	63.8 (23–81)	PCNSL	chemotherapy + RT	na
Niiyama-Uchibori Y (59)	2024	Japan	R	102	44 (43%)	80 (75–92)	DLBCL	R-CHOP, R-CHP, R-CVP	16 (16%)

BL, Burkitt lymphoma; DLBCL, diffuse large B cell lymphoma; FL, follicular lymphoma; HL, Hodgkin lymphoma; HSCT, hematopoietic stem cell transplantation; M, male; MCL, mantle cell lymphoma; na, not available; P, prospective; PCNSL, primary central nervous system lymphoma; R, retrospective; RT, radiotherapy; T-LBL, T-cell lymphoblastic lymphoma.

**Table 2:** The principal technical characteristics of articles published about sarcopenia in lymphoma.

First Author	Imaging method	Parameters evaluated	SMI threshold	Outcome survival
Camus V (22)	Muscle assessment at the L3 level using CT images	SMI	55.8 cm^2^/m^2^ for men 38.9 cm^2^/m^2^ for women	Statistically significant correlation with OS
Lanic H (23)	Muscle assessment at the L3 level using CT images	SMI	55.8 cm^2^/m^2^ for men 38.9 cm^2^/m^2^ for women	Statistically significant correlation with OS and PFS
Caram MV (24)	Muscle assessment at the L3 level using CT images	Total psoas area; total psoas index; lean psoas area; lean psoas index	100 unit decrease (mm^2^/m^2^)	Statistically significant correlation with readmission days and number of complications
Nakamura N (25)	Muscle assessment at the L3 level using CT images	SMI	47.1 cm^2^/m^2^ for men 34.4 cm^2^/m^2^ for women	Not statistically significant correlation with OS and PFS in whole population. Statistically significant correlation with PFS in men
Xiao DY (26)	Muscle assessment at the L3 level using CT images	SMI	53 cm^2^/m^2^ for men 41 cm^2^/m^2^ for women	Statistically significant correlation with probability of toxicities. Not Statistically significant correlation with OS
Xiao DY (27)	Muscle assessment at the L3 level using CT images	SMI	53 cm^2^/m^2^ for men 41 cm^2^/m^2^ for women	na
Go S (28)	Muscle assessment at the L3 level using CT images	SMI	53 cm^2^/m^2^ for men 41 cm^2^/m^2^ for women	Statistically significant correlation with response to treatment, OS, PFS and risk of toxicities
Karmali R (29)	Muscle assessment at the L3 level using CT images	SMI; CXI	55.8 cm^2^/m^2^ for men 38.9 cm^2^/m^2^ for women	No statistically significant correlation with OS and PFS
Chu MP (30)	Muscle assessment at the L3 level using CT images	SMI; SMD	nr	Statistically significant correlation with OS and PFS
Go S (31)	Muscle assessment at the pectoralis muscles and L3 level using CT images	L3-SMI; PM-SMI	52.4 cm^2^/m^2^ for men (L3) 38.5 cm^2^/m^2^ for women (L3) 4.4 cm^2^/m^2^ for men (PM) 3.1 cm^2^/m^2^ for women (PM)	Statistically significant correlation with response to treatment, OS, PFS and risk of toxicities
Jabbour J (32)	Muscle assessment at the L3 level using CT images	SMI	52.4 cm^2^/m^2^ for men 38.5 cm^2^/m^2^ for women	Statistically significant correlated with OS and PFS
DeFilipp Z (33)	Muscle assessment at the L3 level using CT images	SMI	55 cm^2^/m^2^ for men 39 cm^2^/m^2^ for women	Statistically significant correlation with probability of toxicities. No statistically significant correlation with OS
Burkart M (34)	Muscle assessment at the L3 level using CT or CT of PET images	SMI	56.8 cm^2^/m^2^ for men 47.4 cm^2^/m^2^ for women	Statistically significant correlation with OS and PFS in male
Go S (35)	Muscle assessment at the pectoralis muscles and L3 level using CT images	L3-SMI; PM-SMI	52.4 cm^2^/m^2^ for men (L3) 38.5 cm^2^/m^2^ for women(L3) 4.4 cm^2^/m^2^ for men (PM) 3.1 cm^2^/m^2^ for women (PM)	Statistically significant correlation with response to treatment, OS, PFS and risk of toxicities
Lin RJ (36)	Muscle assessment at the L3 level using CT or CT of PET images	SMI	43 cm^2^/m^2^ for men with BMI < 25 41 cm^2^/m^2^ for women 53 cm^2^/m^2^ for men with BMI > 25	Statistically significant correlation with OS and PFS
Mishra S (37)	Muscle assessment at the T4 and L3 level using CT images	SMI	52.4 cm^2^/m^2^ for men 38.5 cm^2^/m^2^ for women	na
Rier HN (38)	Muscle assessment at the L3 level using CT images	SMI	no cutoff suggested	No statistically significant correlation with OS and PFS
Armenian SH (39)	Muscle assessment at the L3 level using CT or CT of PET images	SMI	43 cm^2^/m^2^ for men with BMI < 25 41 cm^2^/m^2^ for women 53 cm^2^/m^2^ for men with BMI > 25	No statistically significant correlation with OS
Bas V (40)	Muscle assessment at the L3 level using CT images	HU; MTV	No cutoff suggested	na
Lucjanic M (41)	Muscle assessment at the L3 level using CT images	SMI	5.82 cm^2^/m^2^	Statistically significant correlation with PFS
Hirota K (42)	Psoas muscle assessment at the L3 level using CT images	PMI; VFA; SFA	6.36 cm^2^/m^2^ for men 3.92 cm^2^/m^2^ for women	na
Guo J (43)	Muscle assessment at the L3 level using CT images	SMA; SMD; SMI; SMG	SMI 27.55 cm^2^/m^2^ SMD 36.86 cm^2^/m^2^ SMG 1462	Statistically significant correlation with OS and probability of toxicities
Iltar U (44)	Psoas muscle assessment at the L3 level using CT images	PMI	4.4 cm^2^/m^2^ for men 3.1 cm^2^/m^2^ for women	Statistically significant correlation with response to treatment, OS and PFS
Besutti G (45)	Muscle assessment at the L3 and proximal thigh level using CT of PET images	SMI; SMD	43 cm^2^/m^2^ for men with BMI < 25 41 cm^2^/m^2^ for women 53 cm^2^/m^2^ for men with BMI > 25 + 52.4 cm^2^/m^2^ for men 38.5 cm^2^/m^2^ for women	SMI not significantly correlated with OS and PFS SMD significantly correlated with OS and PFS
Zilioli V (46)	Muscle assessment at the L3 level using CT or CT of PET images	SMI	55 cm^2^/m^2^ for men 39 cm^2^/m^2^ for women + 47.1 cm^2^/m^2^ for men 34.4 cm^2^/m^2^ for women + 45 cm^2^/m^2^ for men	Statistically significant correlation with OS and PFS in male
Albano D (47)	Muscle assessment at the L3 level using CT of PET images	SMI, ΔSMI	55 cm^2^/m^2^ for men 39 cm^2^/m^2^ for women	Statistically significant correlation with OS and PFS
Albano D (48)	Muscle assessment at the L3 level using CT of PET images	SMI	53 cm^2^/m^2^ for men 45.6 cm^2^/m^2^ for women	Statistically significant correlation with PFS, not with OS
Tan X (49)	Muscle assessment at the L3 level using CT of PET images	SMI	44.7 cm^2^/m^2^ for men 32.5 cm^2^/m^2^ for women	No statistically significant correlation with PFS and OS
Penichoux J (50)	Muscle assessment at the L3 level using CT images	SMI	55.8 cm^2^/m^2^ for men 38.9 cm^2^/m^2^ for women	No statistically significant correlation with PFS and OS
Go S-I (51)	Muscle assessment at the L3 level using CT images	SMI	52.4 cm^2^/m^2^ for men 38.5 cm^2^/m^2^ for women	Statistically significant correlation with OS and PFS
Liao PH (52)	Muscle assessment at the L3 level using CT images	SMI	43 cm^2^/m^2^ for men with BMI < 25 41 cm^2^/m^2^ for women 53 cm^2^/m^2^ for men with BMI > 25	Statistically significant correlation with OS and PFS
Aleixo GFP (53)	Muscle assessment at the L3 level using CT images	SMI; SMD, HU	52 cm^2^/m^2^	No statistically significant correlation with PFS and OS
Chen Y (54)	Muscle assessment at the L3 level using CT images	SMI	44.7 cm^2^/m^2^ for men 32.5 cm^2^/m^2^ for women	Statistically significant correlation with OS and PFS
Rejeski K (55)	Muscle assessment at the L3 level using CT images	SMI, PMI	34.5 cm^2^/m^2^ SMI 4.7 cm^2^/m^2^ PMI	Statistically significant correlation with OS and PFS
Sumransub N (56)	Muscle assessment at the L3 level using CT or CT of PET images	SMI	52.4 cm^2^/m^2^ for men 38.5 cm^2^/m^2^ for women	Statistically significant correlation with PFS, not with OS
Tan X (57)	Muscle assessment at the L3 level using CT of PET images	SMI, ΔSMI	44.7 cm^2^/m^2^ for men 32.5 cm^2^/m^2^ for women	Statistically significant correlation with OS and PFS
Surov A (58)	Muscle assessment at the L3 level using CT images	SMI	52.4 cm^2^/m^2^ for men 38.5 cm^2^/m^2^ for women	Statistically significant correlation with treatment response and risk of toxicities
Niiyama-Uchibori Y (59)	Muscle assessment at the L3 level using CT images	SMI, PMI, ESMI	40.31 cm^2^/m^2^ for men 30.88 cm^2^/m^2^ for women	Statistically significant correlation with OS and PFS

BMI, body mass index; CXI, cachexia index; ESMI, erector spinae muscle index; HU, Hounsfield; L3, third lumbar vertebra; MTV, metabolic tumor volume; OS, overall survival; PET, positron emission tomography; PMI, psoas muscle index; PM, pectoral muscle; PFS, progression free survival; SMI, skeletal muscle index; SMD, skeletal muscle density; SMG, skeletal muscle gage; T4, fourth thoracic vertebra.

In addition to CT, interesting data is emerging about the role of MRI in measuring skeletal muscle mass in extranodal natural killer/T cell lymphoma and PCNSL (61–63). In these studies, sarcopenia parameters were measured at the temporal muscle or masticatory muscle deriving different thresholds (5.5 cm^2^/m^2^), but the findings are only preliminary and need more solid evidence. CT is typically favored over MRI due to its broad availability, lower cost, and faster processing time.

In conclusion, although the studies are heterogeneous, primarily retrospective, and show considerable variability in sample size, it can be argued that sarcopenia measurement using CT (both high and low dose) is a reliable and safe method, often correlated with prognosis. While the overall findings on sarcopenia-related imaging features in lymphoma are promising, the technical challenges and lack of international consensus on defining sarcopenia thresholds impact the widespread adoption of this parameter in clinical practice.

### 2.4 Definition and background

Multiple quantitative metrics obtained from baseline PET/CT, such as metabolic tumor volume (MTV) and total lesion glycolysis (TLG), appear to be viable indicators across different lymphoma subtypes. Specific studies indicate that a heightened MTV value serves as an effective predictor of worse outcomes in lymphoma patients (64–66). Enhanced prognostic models may be developed by integrating baseline MTV or TLG with early responses observed in interim PET/CT scans. Although the notion of MTV is fundamentally straightforward, as it denotes the viable tumor burden, the methodology for its measurement remains a subject of debate (67).

### 2.5 Technical characteristics

Many methodologies have been introduced to quantify MTV and implemented in specific individuals diagnosed with diverse lymphoma subtypes. This has led to multiple cut-offs for MTV that distinguish between favorable and unfavorable prognostic groupings. To date, the most common technique for assessing disease MTV involves the semi-automatic delineation of lesions, namely regions exhibiting abnormal uptake above a predefined threshold (usually 4.0) or percentage of the SUVmax of the most active lesion (typically 41% as suggested by EANM guidelines). Furthermore, several software applications for the automated segmentation of PET scans are being developed to reduce interobserver variability and enhance measurement reproducibility. Another issue hampering the employment of MTV in clinical practice relies on how it is evaluated, since most studies available in the literature use it as a categorical variable, although biomarkers predict outcomes better as continuous variables (68). Currently, it is not yet clear if used MTV as categorical or continuous variable. The standardization of the methodology for MTV measurement and its settling in prediction models are essential to evaluating the potential significance of this variable in the risk classification of lymphoma patients and utilizing it as a prognostic factor in clinical practice. Recently, Boellaard et al. (69) proposed an international benchmark for total metabolic tumor volume measurement in baseline 2-[18F]FDG PET/CT using an automatic segmentation method and a predefined threshold for SUV equal to 4.0 in order to solve any discrepancy between different readers. However, the studied that compared the prognostic role of different threshold for the measurement of MTV demonstrated similar performances (67). TLG is defined as the product of the mean SUV and MTV and has the role to assess the entity of uptake normalized for the tumor burden. Since TLG is a parameter derived from the calculation of MTV, its value is subject to the same issues mentioned for disease volume assessment.

### 2.6 Main results in lymphoma

Concerning the employment of MTV and TLG in the prognosis prediction in adult lymphoma patients, a meta-analysis from Guo et al. (70) reported that a high MTV and TLG significantly predicted shorter overall survival and progression-free survival in different subgroup analyses, including DLBCL, FL, ENKL and HL patients (Table 3). In this analysis, despite MTV and TLG being predictors of prognosis, MTV showed more gleaming results, suggesting that tumor burden is a more reliable instrument for risk stratification irrespective of the entity of glucose consumption. This meta-analysis makes one concept clear, MTV is a good outcome predictor in DLBCL and other lymphoma subtypes, regardless of the measurement method. However, the above-mentioned meta-analysis accounts for not negligible limitations since nearly all the included studies were retrospective and included patients with different lymphoma subtypes submitted to different therapeutic protocols (70). The MTV prognostic value was confirmed in a large DLBCL patient cohort treated with obinutuzumab or R-CHOP in the Phase three REMARC trial, which calculated a MTV cut-off of 220 cm^3^ to identify patients with higher-risk patients (71). Moreover, in a recent retrospective study by Mikhaeel et al. (72) MTV was identified as an optimal parameter to predict OS and PFS in DLBCL patients; as the other studies mentioned, MTV was a preferable variable rather than TLG to predict patients’ prognosis. Furthermore, the authors observed that MTV could predict prognosis independently from the IPI score. A crucial step toward the inclusion of MTV among the parameters for risk assessment in DLBCL patients in clinical practice was performed by the colleagues of the PETRA consortium, who developed the “International Metabolic Prognostic Index” (IMPI), which considered for its calculation age, stage and MTV in a staging setting (72). The first innovation brought about by this study was that MTV was not evaluated as a dichotomic variable but was instead assessed as a continuous variable. The study included DLBCL patients from five different clinical trials, and compared this novel index with the currently utilized IPI, revealing that IMPI could outperform IPI and could enable individualized estimates of patient’s outcome. More recently, different colleagues tried to employ the IMPI in clinical settings other than staging. Winkelmann et al. (73) tried to use the IMPI as a prognosis predictor in patients with relapse or refractory DLBCL undergoing chimeric antigen receptor T-cell therapy (CART), observing a good prediction of PFS; nevertheless, in this casuistry, IMPI had not a significant association with OS (of note, neither IPI did). Moreover, IMPI was tested in patients undergoing immunotherapy with oncastuximab tesirine in relapsed/refractory DLBCL patients, and, despite being significant predictor of PFS and OS, it showed an inferior predictive performance compared to MTV alone (19, 74). Although the IMPI has brought several innovations regarding risk classification in patients diagnosed with DLBCL, it is imperfect in predicting prognosis and may need revisions. Indeed, Michaud et al. (75) tested IMPI in a cohort of DLBCL patients undergoing risk-adapted immunochemotherapy regimen and observed that this novel index slightly overestimated the recurrence rate in their cohort, whereas baseline MTV was a significant predictor of PFS alongside ΔSUVmax and Deauville score. Finally, one recent study found a significant correlation of circulating tumor DNA with MTV, TLG and texture features at diagnosis, suggesting a potential interaction between these parameters (76).

**Table 3:** Summary of main publications about MTV and TLG role in lymphoma.

Author (ref.)	Year	Study design	No. patients	Lymphoma subtype	Clinical setting	Segmentation method	MTV cutoff (cm^3^)	TLG cutoff	Main outcomes
Tseng (84)	2012	R	30	HL	Staging	Visual assessment	/	/	Quantitative interpretation of FDG-PET is a valuable tool to guide the functional imaging for Hodgkin’s disease.
Kim (64)	2013	R	20	NK/T-cell	Staging	SUV > 3.0	14.4 cm^3^	52.7	High MTV adjusted for the IPI score was the best predictor for OS and PFS
Song (85)	2013	R	127	HL	Staging	SUV > 2.5	198	/	MTV was valuable for predicting the prognosis in patients with early stage HL.
Kanoun (65)	2014	R	59	HL	Staging	41% SUVmax	225	/	MTV was independent predictor of PFS
Ceriani (66)	2015	P	103	PMBCL	Staging	25% SUVmax	703	5,814	In univariate analysis, elevated MTV and TLG were significantly associated with worse PFS and OS. Only TLG retained statistical significance for both OS and PFS in multivariate analysis.
Cottereau (81)	2018	P	159	FL	Staging	41% SUVmax	510	/	Baseline MTV better stratified the response to treatment assessed by end-of-induction PET in FL patients.
Zhou (82)	2019	R	84	FL	Staging	41% SUVmax	180	1,364	Baseline TLG was an independent predictor of PFS and OS in FL.
Liang (83)	2019	R	48	FL	Staging	SUV > 2.0 SUV > 2.5 SUV > 3.0	SUV2: 505 SUV2.5: 391 SUV3: 476	SUV2: 3260 SUV2.5: 3080 SUV3: 2677	MTV and TLG were independent predictors of PFS and OS in FL patients.
Vercellino (71)	2020	P	360	DLBCL	Staging	41% SUVmax	220	/	High MTV at baseline was significantly associated with inferior PFS and OS in patients receiving either lenalidomide maintenance or placebo.
Mikhaeel (72)	2022	R	1,241	DLBCL	Staging	SUV > 4.0	Continuous variable	/	IMPI outperformed IPI as prognostic index in DLBCL patients
Winkelmann (73)	2023	P	39	DLBCL	Relapse/ refractory undergoing CART	SUV > 4.0	Continuous variable	/	IMPI had superior prognostic value compared to IPI for the estimation of PFS. IMPI could not predict OS.
Michaud (75)	2023	R	166	DLBCL	Staging	41% SUVmax	510	/	MTV could improve risk stratification of patients undergoing risk-adapted chemotherapy.
Alderuccio (74)*	2024	R	138	DLBCL	Relapse/refractory	SUV > 4	96	926	Both IMPI and dichotomized MTV could predict PFS, however MTV alone had better results than IMPI.
Duffles (76)	2024	R	27	DLBCL	Staging	n.a.	/	/	MTV and TLG values had correlation with circulating tumor DNA.
Yang (80)	2024	R	270	FL	Staging	SUV > 2.5	111.6	141.5	MTV and TLG may provide prognostic value and help to improve the decision-making of initial treatment plans for newly diagnosed FL patients.
Yadgarov (86)	2024	R	115	Pediatric HL	Staging	SUV > 2.5 41% SUVmax	SUV2.5: 160 41%SUV: 143	SUV2.5: 1360 41%SUV: 750	Both MTV and TLG from baseline and interim FDG-PET scans are strong prognostic indicators for treatment response and PFS in pediatric HL

^*^Alderuccio et al. (74) used MTV both as a dichotomized and continuous variable. DLBCL, diffuse large B cell lymphoma; FDG, fluorodeoxyglucose; FL, follicular lymphoma; HL, Hodgkin lymphoma; IMPI, International Metabolic Prognostic Index; IPI:International Prognostic Index; MTV, metabolic tumor volume; NHL, non-Hodgkin lymphoma; NK, natural killer cell; OS, overall survival; R, retrospective; P, prospective; PFS, progression-free survival; PMBCL, primary mediastinal B cell lymphoma; SUV, standard uptake value; TLG, total lesion glycolysis.

As for DLBCL, MTV and TLG might be a useful instrument for prognosis prediction and risk assessment also in FL. Being FL an indolent lymphoma subtype, no conclusive survival advantage has been established for the early commencement of rituximab or chemotherapy (77); subsequently, the watch-and-wait strategy continues to be an essential management choice in FL patients. Consistent with the survival statistics from a prospective randomized clinical trial, approximately half of the follicular lymphoma patients in the watchful waiting group did not require therapy after 3 years (77). In order to select patients needing treatment, the GELF criteria and NCCN guidelines utilize indicators such as patient symptoms, potential organ damage, severe cytopenia, and tumor burden assessment (bulky disease, involvement of at least three lymph nodes each measuring ≥3 cm in diameter), and splenomegaly, for the selection of patients requiring early treatment (78, 79). In this context, MTV and TLG were deemed reliable prognostic factors in patients undergoing therapy and in those in watch-and-wait (80–83). In this setting, more extensive multicentre trials are needed to assess if MTV (expressed as a dichotomic or continuous variable) might be a prognostic factor guiding the starting of treatment, irrespectively of the number of lesions detected. As for NHL, high values of MTV and TLG were found to negatively impact the prognosis also in HLs. In a prior investigation of HL patients undergoing routine regimens, the mean tumor load, adjusted for body surface area using CT measurements, proved to be a predictor of survival compared to the other prognostic models. Determining the metabolic volume of a tumor may be the most effective method for predicting response and its durability. Multivariate analysis conducted by Song et al. (84), including 127 early-stage HL patients treated, showed that high MTV was independently correlated with PFS and OS. In a separate single-centre investigation, Kanoun et al. (65) determined that pre-treatment MTV was a prognostic indicator of patient outcomes in a group of 59 HL patients. This study revealed that patients with low MTV exhibit superior PFS compared to those with high MTV. Multivariate analysis identified baseline total MTV and the reduction in SUVmax in response-assessment PET as the sole independent predictors of PFS, while tumor bulk was not a significant predictor. However, it is noteworthy that, in a similar analysis, Tseng et al. (85) tried using baseline MTV to predict PFS without any significant results. Subsequently, focus has shifted from conventional risk classification methods using baseline PET characteristics to employing interim PET data for guiding early therapeutic adjustments. Several studies measured MTV, TLG, and their temporal variations to assess their potential role as prognosis predictors. For example, Tseng et al. (85) observed that the ratio of MTV values collected in baseline and interim PET was predictive of PFS at 50 months. In a recent study, Yadgarov et al. (86) tested MTV and TLG as prognosis predictors in pediatric HL patients and observed that MTV and TLG were significantly associated with shorter PFS and had a strong correlation with post-treatment Deauville scores.

MTV and TLG showed positive impact also in predicting prognosis in patients that received CAR-T cell therapy as published in this recent meta-analysis (87).

In conclusion, MTV and TLG can be considered two parameters that precisely quantify the tumor burden and its metabolism in lymphoproliferative diseases. Their potential to predict, within certain limits, the treatment outcome in most lymphoma variants is a fascinating area of research. While attempts have already been made to integrate these values into risk stratification models, further studies are needed to make their measurements reproducible and provide the clinician with reliable data to select the best treatment option. Table 1 reports the main findings of the cited articles testing MTV and TLG as prognosis predictors in different lymphoma subtypes and clinical settings ordered by year of publication.

## 3 Dissemination features: Dmax

### 3.1 Definition and background

**Figure 2:**
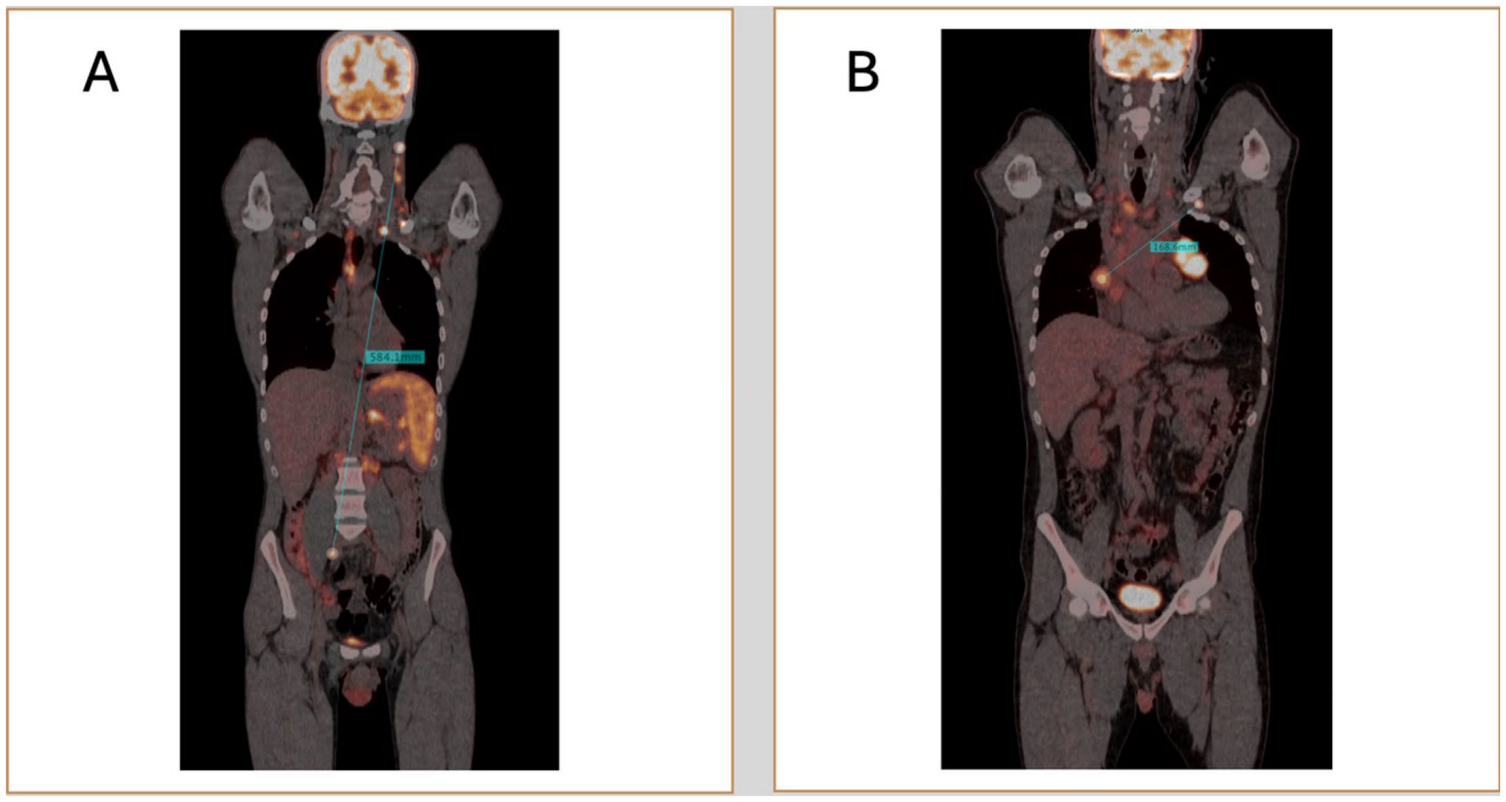
Two representative cases of patients with high **(A)** and low Dmax **(B)**.

With advancements in PET/CT image processing and post-processing software, new opportunities have emerged for the precise and quantitative evaluation of lymphoma. One promising biomarker is Dmax, which measures the maximum tumor dissemination by calculating the distance between the two farthest hypermetabolic lesions detected on PET scans (88, 89). Traditional staging systems, such as the Ann Arbor classification, categorize lymphoma based on the extent of disease spread in a qualitative manner (7). In contrast, Dmax could offer a more precise, quantitative measure of disease dissemination, capturing details that the Ann Arbor system may not fully reflect. The transition from qualitative approaches to more personalized, data-driven quantitative approach could enhance risk stratification, paving the way for more precise prognostics scores (Figure 2).

### 3.2 Technical characteristics

Most studies on Dmax have focused on diffuse large B-cell lymphoma (DLBCL), the most common form of non-Hodgkin lymphoma (Table 4). Prognostication is critical in DLBCL due to its clinical heterogeneity. The concept of Dmax was first introduced by the French group led by Cottereau et al. (90). They demonstrated that Dmax was significantly associated with progression-free survival (PFS) and overall survival (OS) in DLBCL patients. They further showed that combining Dmax with metabolic tumor volume (MTV) provided better risk stratification than using Dmax alone. In a larger study involving 290 patients (91), they confirmed that both Dmax and its normalized form (SDmax), adjusted for body surface area, were strongly correlated with PFS and OS, again suggesting that combining Dmax with MTV enhances prognostic accuracy. Another publication (92) supported these findings in a study involving 382 patients, highlighting the improved risk stratification achieved by combining Dmax with MTV. Similarly, Xu et al. (93) explored the prognostic role of Dmax combined with MTV to stratify risk in patients with low and high-risk categories according to the NCCN-IPI.

**Table 4:** Summary of studies on Dmax in diffuse large B-cell lymphoma (DLBCL).

Author (ref)	Year	Sample size	Dmax cut-off	Type of study	Median age (Range)	M:F	Combination with other parameters	Software/Method used	Principal findings
Cottereau (90)	2020	95	58 cm	Retrospective	46(18–59)	53:42	Dmax + MTV	LIFEx Software	Significantly associated with PFS and OS; combining with TMTV improved risk stratification
Cottereau (91)	2021	290	32 cm	Retrospective	na (60–80)	170:120	SDmax + MTV	LIFEx Software	SDmax sgnificantly associated with PFS and OS; combination with MTV improved patient stratification
Girum et (92)	2022	382	59 cm	Retrospective	62.1* (34–73)	207:175	Dmax + MTV	LIFEx Software	Significantly associated with PFS and OS.
Xu (93)	2023	113	31 cm	Retrospective	61?	57:56	Dmax + MTV	NA	Dmax was associated with PFS.
Eertink (94)	2022	317	cm	Prospective	65(23–80)	161:156	Dmaxbulk + Other Metabolic Parameters	RaCaT	Best predictor of PFS
Eertink (95)	2023	296	Not specified	Prospective	65 (55–72)	152:144	Different dissemination features	RaCaT	Associated with PFS and OS.
Ceriani (96)	2022	240	na	Retrospective	na	119:121	SDmax +Baseline Radiomic Features	LIFEx Software	Part of a predictive radiomics model for OS and PFS.
Dang et (97)	2023	154	53.2 cm	Retrospective	56 (16–87)	78:76	Dmax + %ΔSUVmax	LIFEx Software	Predictive of PFS
Jo (98)	2023	63	27.5 cm	Retrospective	57.3* (21–87)	28:35	Dmax + End-of-Treatment PET	LIFEx Software	Predictor of TTP
Marchal (99)	2023	56	15 cm	Retrospective	60.2*	36:20	SDmax	LIFEx Software	Associated with OS; No association with PFS

*mean; PFS, Progression free survival: OS, Overall survive; TTP, time to progression; MTV, metabolic tumor volume; NA, not available.

For the first time in 2022 the concept of Dmaxbulk was introduced (94, 95) and it was defined as the maximal distance between the largest lesion and any other lesion, which emerged as a strong predictor of treatment outcomes in DLBCL, particularly when combined with other metabolic parameters, improving the positive predictive value (PPV) by 15%. They further validated the prognostic value of Dmax in 2023, showing that baseline radiomic features, including Dmax, were significantly associated with PFS and OS in aggressive B-cell lymphoma. This evidence was confirmed by a subsequent research (96). Dang et al. (97) investigated the combination of baseline Dmax with %ΔSUVmax after 3–4 cycles of immunochemotherapy, finding that this combination improved the predictive efficacy for PFS. Jo et al. (98) extended these findings by showing that Dmax was associated with time to progression (TTP) when combined with end-of-treatment PET scans.

In a recent study Marchal et al. (99), demonstrated that pre-CAR-T cell infusion Dmax was an independent prognostic factor for OS in 56 DLBCL patients but did not impact PFS. These DLBCL studies consistently highlight the value of Dmax as a prognostic tool, particularly when combined with other PET-derived features like MTV. However, a major limitation across studies is the use of varying Dmax cut-offs, which complicates direct comparisons. Future research should focus on standardizing Dmax cut-offs in DLBCL and developing automated methods for calculating this parameter to improve clinical utility.

### 3.3 Main results in lymphoma

#### 3.3.1 Hodgkin lymphoma (HL)

Hodgkin lymphoma (HL) is a highly curable malignancy with modern therapies, but early identification of high-risk patients remains crucial. Five studies have evaluated Dmax in the context of HL, with generally consistent findings (Table 5). Weisman et al. (100) found that Dmax exhibited moderate reproducibility between automated software and physician measurements in pediatric Hodgkin lymphoma patients. While Dmax was associated with outcomes, the study underscored the importance of reproducibility in its measurements for clinical adoption. Driessen et al. (101) also investigated Dmax reproducibility in adult patients with classical Hodgkin lymphoma (cHL), finding high reproducibility, which supports Dmax as a robust biomarker despite heterogeneous measurement methods.

**Table 5:** Summary of studies on Dmax in Hodgkin lymphoma (HL) and other lymphoma subtypes.

Study	Year	Lymphoma subtype	Sample size	Dmax cut-off	Type of study	Median age (years)	M:F	Combination with other parameters	Software/method used	Principal findings
Weisman (100)	2020	Pediatric HL	100	NA	Retrospective	15.8 (5.2–21.4)	60:40	na	Deepmedic	Moderate reproducibility of Dmax measurements between software and physicians
Driessen (101)	2022	cHL (Adults)	105	na	Retrospective	30 (13–66)	47:58	na	RaCaT	High reproducibility of Dmax measurements across different segmentation methods
Zhou (102)	2021	HL	65	57.4 cm	Retrospective	29 (8–72)	45:20	na	LIFEx	Association with PFS and OS.
Durmo (103)	2022	HL	155	20 cm	Retrospective	na	79:76	Dmax + Interim PET	LIFEx Software	Dmax was predictor of PFS; combination with iPET improved accuracy
Drees (104)	2022	HL	30	na	Retrospective	36* (18–66)	na	Dmax + Blood Markers (EV-miRNA, TARC)	LIFEx Software	Dmax was related to blood markers like EV-miRNA and TARC.
Li (105)	2022	Follicular Lymphoma	126	56.73	Retrospective	53(21–76)	63–63	Dmax + TLG	R	Both Dmax and TLG were associated with PFS
Xie (106)	2023	Peripheral T-Cell Lymphoma (PTCL)	95	65.95	Retrospective	64 (16–84)	59:46	Dmax + Bone Marrow Biopsy	LIFEx Software	Dmax, along with bone marrow biopsy results, was significantly linked to PFS and OS.
Albano (107)	2024	Burkitt Lymphoma	78	?	Retrospective	52* (18–80)	51:27	Dmax + eotPET/CT results, MTV and TLG	LIFEx Software	Dmax and Sdmax were significantly correlated OS and treatment response.
Gong (108)	2022	Angioimmunoblastic T-Cell Lymphoma (AITL)	81	65.7	Retrospective	63	53:28	Dmax + MTV	LIFEx Software	Dmax was tied to PFS and OS;combination with TMTV improved risk stratifacation
Vergote (109)	2022	Mantle Cell Lymphoma (MCL)	75	30 cm SDmax 60 cmDmax	Retrospective	66 (58–72)	62–21	Dmax and MTV	MIM	No correlation for Dmax with PFS and OS

*mean; PFS, Progression free survival; OS, Overall survive: MTV, metabolic tumor volume; TLG, total lesion glycolysis; NA, not available.

Zhou et al. (102) demonstrated that Dmax was significantly associated with both PFS and OS in HL patients, similar to findings in DLBCL studies. A more recent study Durmo et al. (103) expanded on these results, showing that Dmax was significantly associated with PFS, especially when combined with interim PET results. This finding suggests Dmax could be integrated into response-adapted treatment strategies increasingly used in HL management.

Moreover, the relationship between Dmax and blood markers, including extracellular vesicle microRNA (EV-miRNA) and thymus activation-regulated chemokine (TARC) was explored with interesting findings (104). They found a significant association between Dmax and these markers, indicating that Dmax may reflect both tumor burden and the tumor microenvironment.

#### 3.3.2 Other lymphoma subtypes

The role of Dmax in lymphoma subtypes beyond DLBCL and HL is underexplored. Li et al. (105) assessed the prognostic value of Dmax in follicular lymphoma (FL), finding that Dmax and total lesion glycolysis (TLG) were significantly associated with PFS in a population of 126 grade 1-3a FL patients. The report that a scoring system integrating Dmax and TLG performed better (c-index 0.785) was superior to the predictive capability of the conventional scores FLIPI2, and PRIMA-Prognostic Index (C-index: 0.628–0.701). Peripheral T-cell lymphoma (PTCL) is a rare and aggressive lymphoma with a poor prognosis. Xie et al. (106) reported that Dmax, along with bone marrow biopsy results, was significantly associated with both PFS and OS in 95 PTCL patients. Albano et al. (107) showed that Dmax was significantly correlated with OS in adult Burkitt lymphoma patients. Gong et al. (108) found a similar association in Angioimmunoblastic T-cell lymphoma (AITL) patients, with combining Dmax and MTV enhancing risk stratification, echoing results from DLBCL and HL studies.

Conversely, in mantle cell lymphoma (MCL) Dmax did not significantly correlate with prognosis, with MTV instead identified as the primary predictor of outcomes (109). This suggests that Dmax’s applicability may vary across lymphoma subtypes, warranting further research to clarify its role in MCL.

Despite the reproducibility of Dmax measurements highlighted in some studies, further automation is needed to ensure consistency and reduce operator dependency. Another challenge is the size and diversity of patient samples: many studies, especially those on rare subtypes like PTCL and AITL, have small cohorts. Large-scale, multicentre studies are needed to validate findings and assess Dmax’s role in broader, more diverse populations. Finally, it is crucial to explore the use of Dmax in patients treated with emerging therapies, such as CAR-T cells or bispecific antibodies. These treatments could alter lymphoma prognostics, making it urgent to evaluate Dmax’s utility in these new therapeutic contexts.

## 4 Radiomics

### 4.1 Definition and background

New radiomic PET parameters emerged for histologic assessment and prognosis prediction depending on the intra-lesion ^18^F-FDG spatial distribution (7, 110, 111). The potential impact of quantitative parameters in the study of hematopoietic malignancies has recently emerged with promising results and revealing some applications such as survival prediction, assessment of bone marrow involvement and differentiation diagnosis between lymphoma and other malignancies. Additionally, artificial intelligence techniques have been utilized in radiomics to forecast factors associated with treatment strategies, such as tumor subtypes, survival rates, and disease recurrence. Predictive models can be developed using multi-parametric radiomic image features to personalize patient decision-making, either independently or in conjunction with established clinical, biological, and laboratory data (111).

### 4.2 Technical characteristics

In 2021, Rizzo et al. (111) completed a systematic review of original papers in the field of PET radiomics in patients with lymphoma, here updated with new papers up to 31 August 2024 (Table 6). About acquisition protocols, most of papers followed he EANM guidelines for PET/CT acquisition protocols (90, 112–137). About volume selection and contouring, semi-automatic method was used in most of studies (90, 112–118, 122–126, 135–137). The investigation included only the largest tumor site in several studies (112, 113, 119–123, 125, 131–134), while other papers considered all lesions (90, 115–118, 124, 126–129). About data collection, all studies considered conventional semi-quantitative PET/CT parameters at least in the first data analysis, most of papers considered histogram features (112–122, 124–128, 130–137) or higher order textural features (112, 114–137). Some papers included in the final analysis all the extracted features (90, 113, 114, 117–120, 126, 128, 131, 133, 136, 137), while the others performed a selection of the significant features with respect to an outcome before building models. Concerning data analysis, most of studies used a linear regression model (90, 112–116, 118, 119, 121–129, 131–135, 137). Only in few cases, the authors split the patients into a training and a separate test group to independently validate the models in few studies (117, 120, 123, 127, 128, 136). Generally, all these studies had different clinical goals.

**Table 6:** Characteristics of the included studies (2014–2020), with different clinical purposes (prognostication, histology and bone marrow involvement).

Author (ref)	Year	Country	Design	Lymphoma type	n	Software	Volume Segmentation	Extracted features	Train/Test	Model	Selected features
Ko KY (112)	2016	Taiwan	retrospective	Nasal type NK/T-cell	17	MATLAB	Semi-automatic, only lesion	SUV, clinical features, HISZE, HIZE, LISZE, busyness, coarness, BWS, RLV	No	Linear	dissimilarity, LISZE
Bouallègue FB (113)	2017	France	retrospective	Bulky HL NHL	57	na	Semi-automatic, one lesion	SUVMax, SUVpeak, SUVmean variance, skewness, kurtosis MTV, TLG, GLCM, shape	No	Linear	All the extracted
Parvez A (114)	2018	Canada	retrospective	NHL	82	LifeX	Semi-automatic, one to three	GLCM, NGLDM, GLRLM, GLZLM, histogram, sphericity	No	Linear	All the extracted
Lue KH (115)	2019	Taiwan	retrospective	HL	35	MATLAB	Semi-automatic, all lesions	SUV, HU, GLRM, GLSZM grey level	No	Linear	different for treatment response, PFS and OS
Lue KH (116)	2019	Taiwan	retrospective	HL	42	MATLAB	Semi-automatic, all lesions	All orders PET features	No	Linear	SUV kurtosis, MTV, INU, RLN, wavelet HLH
Mayerhoefer ME (117)	2019	USA	retrospective	Mantle Cell Lymphoma	107	na	Semi-automatic, all lesions	SUVMax, SUVmean, SUVpeak, TMTV, TTLG, GLCM	Yes	Non linear	TMTV, GLCM entropy
Milgrom SA (118)	2019	USA	retrospective	NHL	251	IBEX, MIM	Semi-automatic, all lesion	entropy, uniformity, skewness, GLCM	No	Linear	All the extracted
Tatsumi M (119)	2019	Japan	retrospective	FL	45	PESTAT	Automatic, one lesion	Homogeneity, entropey, SRE, LRE, LGZE, HGZE	No	Linear	All the extracted
Wang H (120)	2019	China	retrospective	Nasal type NK/T-cell	110	LifeX, LASSO	Semi-automatic, one lesion	SUV, histogram, shape, GLCM, NGLDM, GLRLM, GLZLM.	Yes	Non linear	All the extracted
Wu J (121)	2019	USA	retrospective	DLBCL	45	BTF	Automatic, one lesion	SUV, GLCM GLRLM, GLSIZM Clinical and genomics	No	Linear	SUV-based, gene expression
Zhou Y (122)	2019	China	retrospective	Gastric DLBCL	35	LifeX	Semi-automatic, one lesion	All orders PET features	No	Linear	MTV, kurtosis, volume, sphericity, HGRE, LRHGE, GLNU, RLNU, LZE, HGZE, LZLGE, LZHGE, ZP
Aide N (123)	2020	France	retrospective	DLBCL	132	LifeX	Semi-automatic, one lesion	conventional, GLCM, GLSZM, SZE, LZE, LGZE, SZLGE, SZHGE, SZHGE, LZLGE, LZHGE, GLNU, ZLNU, ZP	Yes	Linear	histogram, LZE, LZGE, LZHGE, GLNU, AS, ZP
Cottereau AS (90)	2020	France	retrospective	DLBCL	95	LifeX	Semi-automatic, all lesions	TMTV, TLG, dissemination	No	Linear	All the extracted
Rodriguez T MG (124)	2020	Uruguay	prospective	Pediatric HL	21	na	Semi-automatic, all lesions	All orders PET features	No	Linear	GLCM and NGTDM
Sun Y (125)	2020	China	retrospective	Gastric GLBC	30	Image analyzer	Manual, one lesion	All orders PET/CT features	No	Linear	SUVMean, frequency, entropy, volume, max diameter, entropy
Wang M (126)	2020	China	retrospective	Primary Renal/adrenal	19	LifeX	Manual, one lesion	Histogram, GLCM, GLRM, NGLDM GLZLM	No	Linear	All the extracted
Lue KH (127)	2021	Taiwan	retrospective	DLBCL	83	OsiriX, LASSO	Semi-automatic, all lesions	All orders PET features	Yes	Linear	GLN, RLN, GLRLM
Lartizien C (128)	2014	France	retrospective	BCL and HL	25	SVM	Manual	First order, GLCM, GLRLM, GLISZM, GLDM	Yes	Linear	Kurtosis_PET_, Haralick coefficients, GLISZ, GLRL
Kong Z (129)	2019	China	retrospective	Central nervous lymphoma	77	Pyradiomics	Manual	SUVmax, MTV, TLG, first order, GLCM GLRLM, GLSZM	No	Linear	First Order, GLCM, GLRLM, GLDM
Lippi M (130)	2019	Italy	retrospective	Different lymphomas	60	CGITA	Manual	All orders PET/CT features	No	Non linear	All orders PET/CT features
Ou X (131)	2019	China	retrospective	Breast lymphoma	44	LifeX	Manual	Histogram, SHAPE, GLCM, GLRLM, NGLDM, GLZLM	No	Linear	All the extracted
Xu H (132)	2020	China	retrospective	Hepatic lymphoma	100	LifeX	Manual	SUV, TLG, HISTO, Shape, GLCM, GLRM, NGLDM, DLZLM	No	Linear	SUV, TLG, shape, GLCM, GLRLM_GLNU, NGLDM_contrast, GLZLM_GLNU
Ou X (133)	2020	China	retrospective	Breast lymphoma	44	LifeX	Manual	Histogram, SHAPE, GLCM, GLRLM, NGLDM, GLZLM	No	Linear	Six different models
Sun YW (134)	2020	China	retrospective	Gastric Lymphoma	79	Image Analyzer 2.0	Manual	Histogram, GLCM	No	Linear	All features extracted
Aide N (135)	2018	France	retrospective	DLBCL	82	LifeX	Semi-automatic	All orders PET features	No	Linear	All features extracted
Mayerhoefer ME (136)	2020	USA	retrospective	Mantle Cell Lymphoma	97	na	Semi-automatic	SUV derived, histogram, GLCM	Yes	Non Linear	All features extracted
Kenawy MA (137)	2020	Egypt	retrospective	na	44	Chang-Gung Image Texture Analysis	Semi-automatic	All orders PET features	No	Linear	All Features extracted

HL, Hodgkin lymphoma; NHL, non-Hodgkin Lymphoma; DLBCL, diffuse large B-cell lymphoma; FL, Follicular Lymphoma; SUV, Standardized Uptake Value; MTV, Metabolic Tumor Volume; TLG, Total lesion glycolysis; HU, Hounsfield unit; GLCM, grey-level co-occurrence matrix; NGLDM, neighborhood grey-level different matrix; GLRLM, grey-level run length matrix; GLZLM, grey-level zone length matrix; GLSZM, grey-level size-zone matrix; HISZE, high-intensity short-zone emphasis; HIZE, high-intensity zone emphasis; HU, Hounsfield unit; LISZE, low-intensity short-zone emphasis; BWS, black-white symmetry; RLV, run-length variability; SRE, Short Run emphasis; LRE, long-run emphasis; LGZE, low grey-level zone emphasis; HGZE, high grey-level zone emphasis; SZE, short zone emphasis; LZE, long zone emphasis; SZHGE, short zone high grey level emphasis; LZHGE, short zone emphasis; LZE, long zone emphasis; LZHGE, long zone high grey level emphasis; GLNU, grey level non uniformity for zone; ZLNU, zone length non uniformity; ZP, zone percentage; na, not available.

### 4.3 Main results in lymphoma

**Figure 3:**
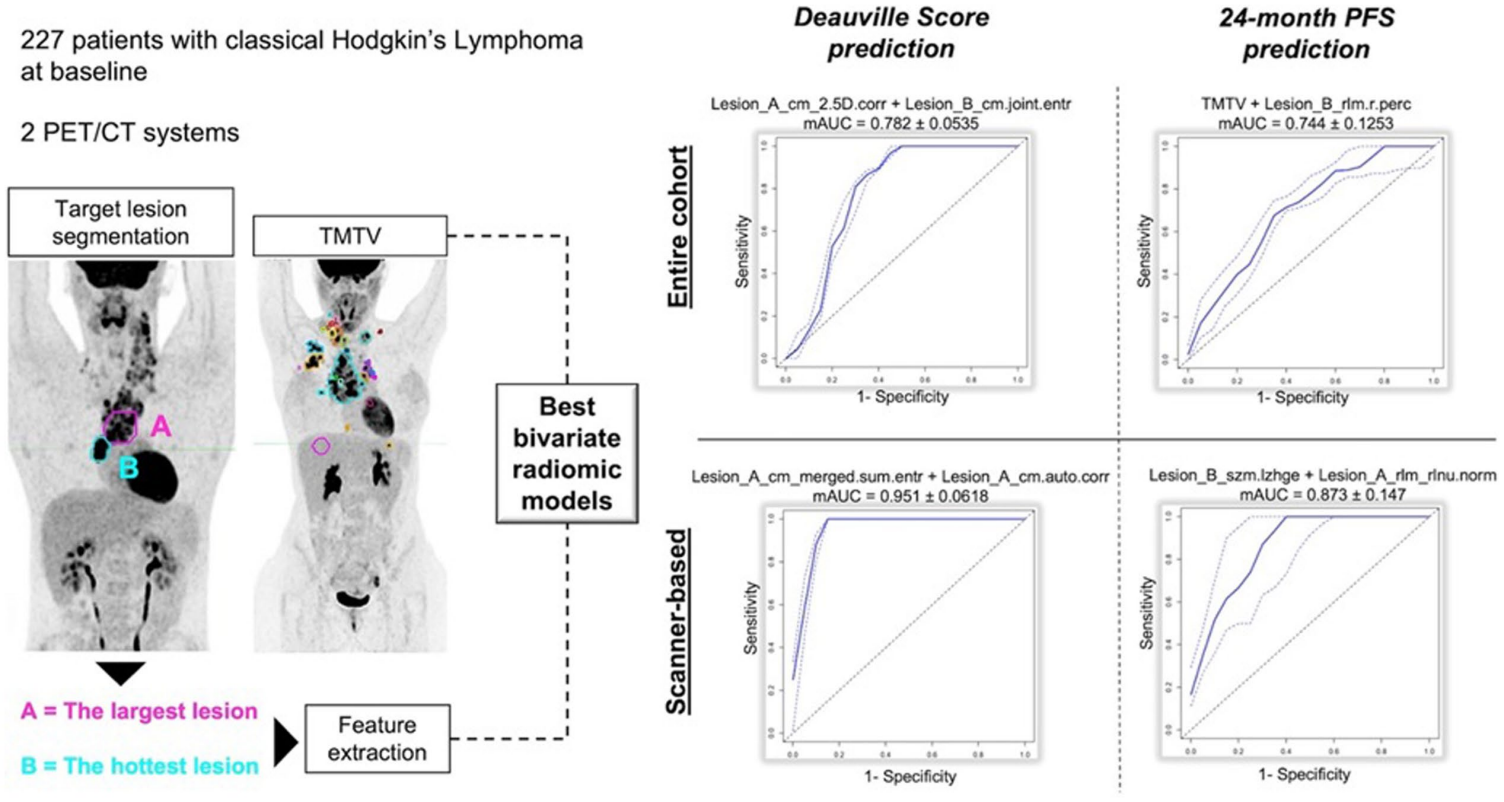
Example of internally validated and retrospective radiomic models applied to PET/CT in Lymphoma, using the most promising image features from Lesion A with largest diameter and Lesion B with highest SUVmax, with regard to Deauville Score (<4 or ≥ 4) and 24-month progression free survival, respectively.

Thus, we decided to divide them in three sub-groups according to clinical purposes: (1) prognosis/outcome; (2) histology and (3) bone marrow involvement researches (Table 6). More common lymphoma subtypes investigated were Hodgkin lymphoma (115, 116, 118, 125), diffuse large B cell lymphoma (90, 121–123, 126, 135), or more than one subtype of lymphoma in the same analysis (113, 114, 125, 127–133, 137). Most of articles aimed to predict outcome, prognosis or survival. Regarding prognostic studies, all papers included in this subgroup revealed a significant association among the radiomic model and patients’ outcome (90, 112–127). However, the radiomic model and lymphoma subtype studied resulted in different patterns of predictive features (imaging, clinical, and/or histopathological) across the various studies (Table 6). All studies examining the ability of radiomic features to distinguish between lymphoma lesions and other malignancies (129–134) or para-physiological sites of FDG uptake (e.g., brown adipose tissue) (128) found a strong correlation between the proposed model and histopathological findings. Similarly, some studies evaluating bone marrow involvement using radiomics demonstrated a significant correlation between the model and the presence of bone marrow involvement (135–137). In Table 7 recent papers published in the time frame 2021–2024 were reported (94, 96, 138–158) (Figure 3). About technical aspects, most of recent papers used semi-automatic volume selection and contouring for radiomic purpose, with an extensive use of all order PET radiomic features (first, second and third order features) (94, 96, 138–151). Moreover, in the last years the use of trained-tested validation systems of the models and non-linear machine-learning methods became more common in scientific literature worldwide (139, 140, 144, 146, 149, 150), with particular regard to academic papers from China. Only few papers started to evaluate multiphase/delta radiomics between baseline and further PET scans (144, 145, 153), or to use external validation cohorts to ensure robust reproducibility of the models (146, 149). About clinical findings, most of recent papers mainly focused on the prognostic use of radiomics (94, 96, 138–155), with particular regard to diffuse large B cell lymphoma (94, 96, 143–147). At the same time, the interest in the use of radiomic feature for histology classification and bone marrow prediction seems to be residual in the last years in scientific literature (156–158). Combined predictive models using both radiomic features of different orders and conventional clinical parameters commonly emerged as the best choice in most of papers (138–140, 146–151). In particular, several radiomic features have been sometimes combined in synthetic radiomics scores, sometimes as a result of machine-learning analysis methods, even though real-world data of those models in routinely context are still missing.

**Table 7:** Update on recent studies (2021–2024), with different clinical purposes (prognostication, histology and bone marrow involvement).

Author (ref)	Year	Country	Design	Lymphoma type	*n*	Software	Volume Segmentation	Extracted features	Train/Test	Model	Selected features
Eertink (94)	2022	Netherlands	retrospective	DLBCL	317	Accurate	Automatic (all lesions)	All orders PET features	No	Linear	Combined models
Ceriani (96)	2022	Switzerland	retrospective	DLBCL	263	PyRadiomics	Automatic (all lesions)	All orders PET features	Yes	Linear	GLCM, GLDM, GLSZM
Jimenez (138)	2022	Unites States	retrospective	miscellaneous	169	MiM	Semi-automatic	All orders PET features	Yes	Linear	Combined models
Frood (139)	2022	UK	retrospective	DLBCL	229	PyRadiomics	Semi-automatic	All order PET features	Yes	Non-linear	Combined models
Jiang (140)	2022	China	retrospective	Gastrointestinal DLBCL	140	PyRadiomics	Semi-automatic	All order PET features	Yes	Non-linear	Combined models
Ortega (141)	2023	Canada	retrospective	HL	88	LifeX	Semi-automatic	All order PET features	No	Linear	GLRLM
Triumbari (142)	2023	Italy	retrospective	HL	227	Moddicom	Semi-automatic (two targets)	All order PET features	Yes	Linear	GLCM
Li (143)	2023	China	retrospective	DLBCL	129	LifeX	Semi-automatic	All order PET features	Yes	Linear	Second order features
Cui (144)	2023	China	retrospective	DLBCL	271	na	na	All order PET features (baseline and after treatment)	Yes	Non-linear	na
Samimi (145)	2023	Iran	retrospective	miscellaneous	126	LifeX	Semi-automatic	All order PET features (dual time point)	Yes	Linear	Second and third order features
Zhao (146)	2023	China	retrospective	DLBCL	240	LifeX	Semi-automatic	All order PET features	Yes (external)	Non-Linear	Combined models
Jing (147)	2023	China	retrospective	DLBCL	201	LifeX	Semi-automatic	All order PET features	Yes	Linear	Combined models
Ligero (148)	2023	Spain	prospective	DLBCL (CAR-T)	93	MiM	Semi-automatic	All order PET features	Yes	Linear	Radiomic scores, Combined models
Driessen (149)	2023	Netherlands	prospective	HL	113	Accurate	Semi-automatic	All order PET features	Yes (external)	Non-linear	Combined models
Carlier (150)	2024	France	prospective	DLBCL	545	PyRadiomics	Semi-automatic	All order PET features	Yes	Non-linear	Combined models
Luo (151)	2024	China	retrospective	Nasal Type NK/T	126	PyRadiomics	Semi-automatic	All order PET features	Yes	Linear	Radiomic scores, Combined models
Albano (152)	2024	Italy	retrospective	Primary Gastric	91	LifeX	Semi-automatic	First order features	No	Linear	Shape sphericity
Yousefirizi (153)	2024	Canada	retrospective	Primary Mediastinal	31	MiM, PyRadiomics	Semi-automatic	First and second order features (delta radiomics)	Yes	Non-linear	Baseline Radiomics
Jing (154)	2024	China	retrospective	DLBCL	126	LifeX	Semi-automatic	All order PET features	Yes	Linear	Combined models
Jing (155)	2024	China	retrospective	DLBCL	239	LifeX	Semi-automatic	na	na	na	na
Zhu (156)	2021	China	retrospective	Renal Lymphoma	21	LifeX	Manual	Histogram, GLCM, GLRM, GLZLM	No	Linear	na
Lovinfosse (157)	2022	Belgium	retrospective	Multiple, Sarcoidosis	420	RadiomiX	Manual	All orders PET features	No	Non-linear	Combined radiomic models
Han (158)	2021	Korea	retrospective	DLBCL	144	Lifex	Manual	All orders PET features	No	Linear	GLZLM

HL, Hodgkin lymphoma; NHL, non-Hodgkin Lymphoma; DLBCL, diffuse large B-cell lymphoma; FL, Follicular Lymphoma; SUV, Standardized Uptake Value; MTV, Metabolic Tumor Volume; TLG, Total lesion glycolysis; HU, Hounsfield unit; GLCM, grey-level co-occurrence matrix; NGLDM, neighborhood grey-level different matrix; GLRLM, grey-level run length matrix; GLZLM, grey-level zone length matrix; GLSZM, grey-level size-zone matrix; HISZE, high-intensity short-zone emphasis; HIZE, high-intensity zone emphasis; HU, Hounsfield unit; LISZE, low-intensity short-zone emphasis; BWS, black-white symmetry; RLV, run-length variability; SRE, Short Run emphasis; LRE, long-run emphasis; LGZE, low grey-level zone emphasis; HGZE, high grey-level zone emphasis; SZE, short zone emphasis; LZE, long zone emphasis; SZHGE, short zone high grey level emphasis; LZHGE, short zone emphasis; LZE, long zone emphasis; LZHGE, long zone high grey level emphasis; GLNU, grey level non uniformity for zone; ZLNU, zone length non uniformity; ZP, zone percentage; na, not available.

## 5 Discussion and conclusions

**Figure 4:**
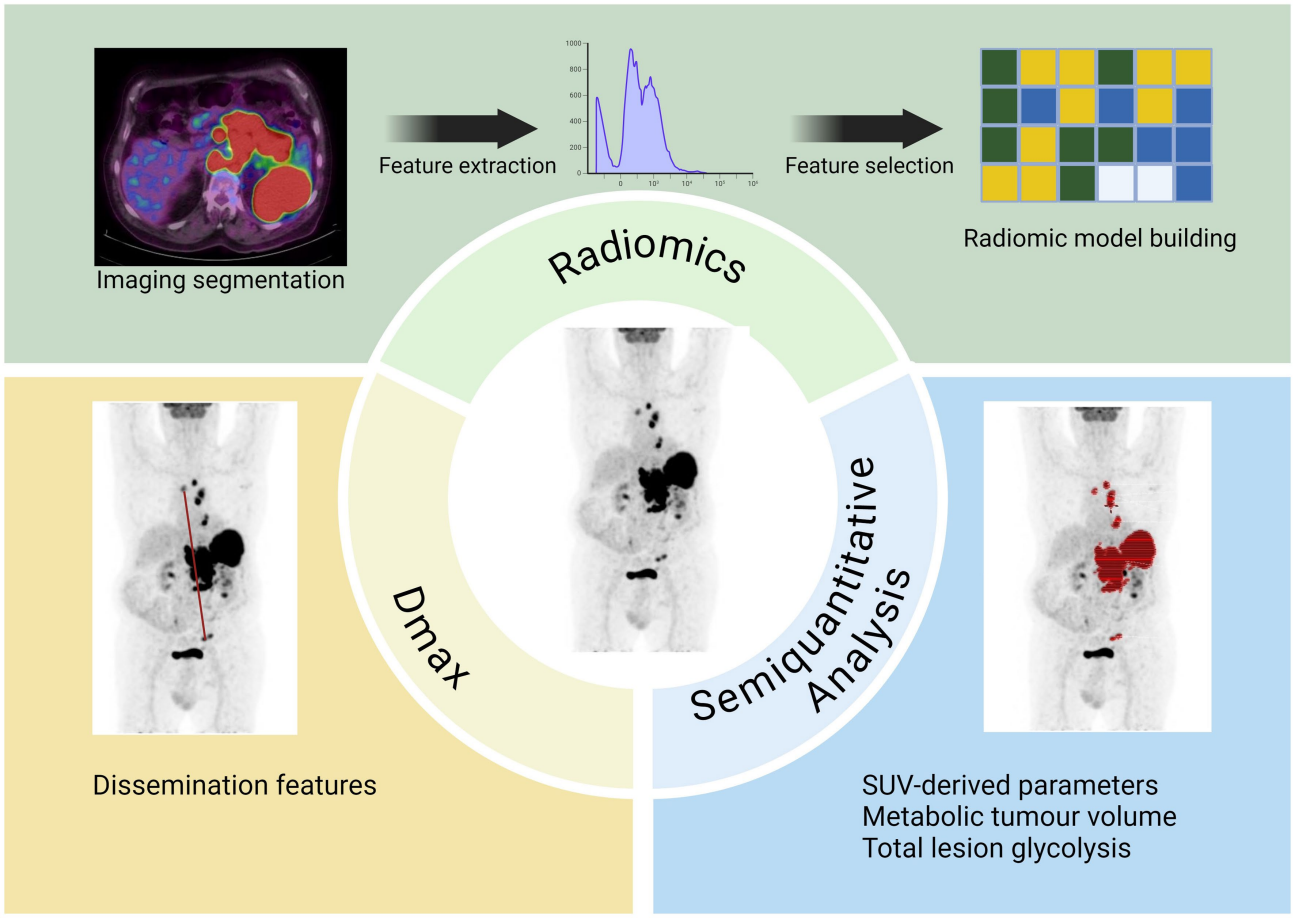
A summary of the potential quantitative PET parameters and their features.

In this review, we focused on the potential role of semiquantitative parameters derived by 2-[^18^F]FDG PET/CT in lymphoma. Despite different functions described, all these variables seem to be promising and effective prognostic factors. However, we have also some limitations such as the retrospective nature of most articles, the relatively small number of patients recruited and the wide heterogeneity of patients included concerning epidemiological and clinical aspects. For these reasons, other investigations on larger populations would be shareable. Besides, many authors analyzed only one kind of features excluding the others and this strongly limits the possibility to exhaustively understand the meaning of these variables. Research including all these features (MTV, TLG; Dmax, SMI and radiomics) could better comprehend the relationship between them and derive combined model to predict prognosis (Figure 4). The integration of these semiquantitative PET-based biomarkers into clinical trials and everyday clinical practice appears imminent; however, several challenges must be addressed before these biomarkers are fully ready for widespread use. For these reasons, visual score or quantitative extension derived in pediatric population, like qPET (159) that utilizes SUVpeak of the residual lesion and average uptake of the liver, are yet utilized.

Furthermore, these interesting parameters still need to be tested in light of the new treatments and new technologies that have been developed. The role of PET-based biomarkers in patients treated with novel agents is still largely uncharted; however, quantitative imaging holds promise for developing risk-adapted treatment strategies for lymphoma patients. The ultimate aim is to create decision-making models that can more accurately identify those who will benefit most from specific therapies. The technological progress in nuclear medicine with the introduction of “new total body” PET scanners could be a significant advantage in this field. Conventional PET/CT systems usually have a 20 cm wide detector ring and the scan takes normally 25–30 min according to the patient height and time for bed position. With the total body scanners detector ring up to 200 cm and a true whole-body PET/CT can be performed in a few minutes with superior image quality compared to current PET/CT systems. Moreover, with these new scanners the uptake detection increases significantly affecting a dramatic change in PET quantification. Moreover, another potential issue is the different acquisition protocols available in every department. It is well known that the application of TOF can impact the image quality and interpretation in the clinical PET data (160), such as also semiquantitative parameters as SUV, MTV and TLG. However, specific studies on lymphoma are lacking.

This review presents some limitations such as the non-systematic nature of this review, selecting arbitrary the articles to include, the long time period of included studies and the heterogeneity of patients included (in terms of disease, technical features, …).

## References

[1] Zanoni L Bezzi D Nanni C Paccagnella A Farina A Broccoli A. PET/CT in non-Hodgkin lymphoma: an update. Semin Nucl Med. (2023) 53:320–51. doi: 10.1053/j.semnuclmed.2022.11.001, PMID: 36522191

[2] Al-Ibraheem A Mottaghy FM Juweid ME. PET/CT in Hodgkin lymphoma: an update. Semin Nucl Med. (2023) 53:303–19. doi: 10.1053/j.semnuclmed.2022.10.006, PMID: 36369090

[3] Albano D Treglia G Gazzilli M Cerudelli E Giubbini R Bertagna F. ^18^F-FDG PET or PET/CT in mantle cell lymphoma. Clin Lymphoma Myeloma Leuk. (2020) 20:422–30. doi: 10.1016/j.clml.2020.01.018 32169480

[4] Albano D Bertagna F Giubbini R. ^18^F-FDG PET/CT role in Burkitt lymphoma. Clin Translat Imaging. (2020) 8:39–45. doi: 10.1007/s40336-020-00356-2

[5] Albano D Durmo R Treglia G Giubbini R Bertagna F. ^18^F-FDG PET/CT or PET role in MALT lymphoma: an open issue not yet solved-A critical review. Clin Lymphoma Myeloma Leuk. (2020) 20:137–46. doi: 10.1016/j.clml.2019.10.006 32029397

[6] Kostakoglu L Chauvie S. PET-derived quantitative metrics for response and prognosis in lymphoma. PET Clin. (2019) 14:317–29. doi: 10.1016/j.cpet.2019.03.002 31084772

[7] Cheson BD Fisher RI Barrington SF Cavalli F Schwartz LH Zucca E. Recommendations for initial evaluation, staging, and response assessment of Hodgkin and non-Hodgkin lymphoma: the Lugano classification. J Clin Oncol. (2014) 32:3059–67. doi: 10.1200/JCO.2013.54.8800, PMID: 25113753 PMC4979083

[8] Barrington SF Mikhaeel NG Kostakoglu L Meignan M Hutchings M Müeller SP. Role of imaging in the staging and response assessment of lymphoma: consensus of the international conference on malignant lymphomas imaging working group. J Clin Oncol. (2014) 32:3048–58. doi: 10.1200/JCO.2013.53.5229, PMID: 25113771 PMC5015423

[9] Alderuccio JP Kuker RA Yang F Moskowitz CH. Quantitative PET-based biomarkers in lymphoma: getting ready for primetime. Nat Rev Clin Oncol. (2023) 20:640–57. doi: 10.1038/s41571-023-00799-2, PMID: 37460635

[10] Rosenberg IH. Sarcopenia: origins and clinical relevance. J Nutr. (1997) 127:990S–1S. doi: 10.1093/jn/127.5.990S, PMID: 9164280

[11] Fielding RA Vellas B Evans WJ Bhasin S Morley JE Newman AB. International working group on sarcopenia. Sarcopenia: an undiagnosed condition in older adults. Current consensus definition: prevalence, etiology, and consequences. J Am Med Dir Assoc. (2011) 12:249–56. doi: 10.1016/j.jamda.2011.01.003, PMID: 21527165 PMC3377163

[12] Cruz-Jentfot AJ Bahat G Bauer J Boirie Y Bruyere O Cederholm T. Sarcopenia: revised European consensus on definition and diagnosis. Age Ageing. (2019) 48:16–31. doi: 10.1093/ageing/afy169 30312372 PMC6322506

[13] Pratesi A Tarantini F Di Bari M. Skeletal muscle: an endocrine organ. Clin Cases Miner Bone Metab. (2013) 10:11–4. doi: 10.11138/ccmbm/2013.10.1.011, PMID: 23858303 PMC3710002

[14] Giudice J Taylor JM. Muscle as a paracrine and endocrine organ. Curr Opin Pharmacol. (2017) 34:49–55. doi: 10.1016/j.coph.2017.05.005, PMID: 28605657 PMC5808999

[15] Shachar SS Williams GR Muss HB Nishijima TF. Prognostic value of sarcopenia in adults with solid tumours: a meta-analysis and systematic review. Eur J Cancer. (2016) 57:58–67. doi: 10.1016/j.ejca.2015.12.030, PMID: 26882087

[16] Albano D Dondi F Ravanelli M Tucci A Farina D Giubbini R. Prognostic role of "radiological" sarcopenia in lymphoma: a systematic review. Clin Lymphoma Myeloma Leuk. (2022) 22:e340–9. doi: 10.1016/j.clml.2021.11.006, PMID: 34893457

[17] Surov A Wienke A. Sarcopenia predicts overall survival in patients with malignant hematological diseases: A meta-analysis. Clin Nutr. (2021) 40:1155–60. doi: 10.1016/j.clnu.2020.07.023, PMID: 32768316

[18] Li Y Sheng Q Li J Liu W Ma L Han L. Sarcopenia is a prognostic factor in lymphoma patients: a systematic review and meta-analysis. Leuk Lymphoma. (2024) 65:1595–608. doi: 10.1080/10428194.2024.2371500, PMID: 39086237

[19] Anabtawi NM Pasala MS Grimshaw AA Kharel P Bal S Godby K. Low skeletal muscle mass and treatment outcomes among adults with haematologic malignancies: a systematic review and meta-analysis. J Cachexia Sarcopenia Muscle. (2024) 15:1084–93. doi: 10.1002/jcsm.13446, PMID: 38558541 PMC11154774

[20] Shen W Punyanitya M Wang ZM Gallagher D Albu J Heymsfield SB. Total body skeletal muscle and adipose tissue volumes: estimation from a single abdominal cross-sectional image. J Appl Physiol. (2004) 97:2333–8. doi: 10.1152/japplphysiol.00744.2004, PMID: 15310748

[21] Mourtzakis M Prado CMM Lieffers JR Reiman T McCargar LJ Baracos VE. A practical and precise approach to quantification of body composition in cancer patients using computed tomography images acquired during routine care. Appl Physiol Nutr Metab. (2008) 33:997–1006. doi: 10.1139/H08-075 18923576

[22] Camus V Lanic H Kraut J Modzelewski R Clatot F Picquenot JM. Prognostic impact of fat tissue loss and cachexia assessed by computed tomography scan in elderly patients with diffuse large B-cell lymphoma treated with immunochemotherapy. Eur J Haematol. (2014) 93:9–18. doi: 10.1111/ejh.12285, PMID: 24520908

[23] Lanic H Kraut-Tauzia J Modzelewski R Clatot F Mareschal S Picquenot JM. Sarcopenia is an independent prognostic factor in elderly patients with diffuse large B-cell lymphoma treated with immunochemotherapy. Leuk Lymphoma. (2014) 55:817–23. doi: 10.3109/10428194.2013.816421 23781925

[24] Caram MV Bellile EL Englesbe MJ Terjimanian M Wang SC Griggs JJ. Sarcopenia is associated with autologous transplant-related outcomes in patients with lymphoma. Leuk Lymphoma. (2015) 56:2855–62. doi: 10.3109/10428194.2015.1014359, PMID: 25739940

[25] Nakamura N Hara T Shibata Y Matsumoto T Nakamura H Ninomiya S. Sarcopenia is an independent prognostic factor in male patients with diffuse large B-cell lymphoma. Ann Hematol. (2015) 94:2043–53. doi: 10.1007/s00277-015-2499-4 26385388

[26] Xiao DY Luo S O’Brian K Sanfilippo KM Ganti A Riedell P. Longitudinal body composition changes in diffuse large B-cell lymphoma survivors: a retrospective cohort study of United States veterans. J Natl Cancer Inst. (2016) 108:djw145. doi: 10.1093/jnci/djw145, PMID: 27381623 PMC5241900

[27] Xiao DY Luo S O’Brian K Ganti A Riedell P Sanfilippo KM. Impact of sarcopenia on treatment tolerance in United States veterans with diffuse large B-cell lymphoma treated with CHOP-based chemotherapy. Am J Hematol. (2016) 91:1002–7. doi: 10.1002/ajh.24465, PMID: 27356783 PMC5324973

[28] Go SI Park MJ Song HN Kim HG Kang MH Lee HR. Prognostic impact of sarcopenia in patients with diffuse large B-cell lymphoma treated with rituximab plus cyclophosphamide, doxorubicin, vincristine, and prednisone. J Cachexia Sarcopenia Muscle. (2016) 7:567–76. doi: 10.1002/jcsm.12115, PMID: 27104110 PMC4833756

[29] Karmali R Alrifai T Fughhi IAM Ng R Chukkapalli V Shah P. Impact of cachexia on outcomes in aggressive lymphomas. Ann Hematol. (2017) 96:951–6. doi: 10.1007/s00277-017-2958-1 28417157

[30] Chu MP Lieffers J Ghosh S Belch A Chua NS Fontaine A. Skeletal muscle density is an independent predictor of diffuse large B-cell lymphoma outcomes treated with rituximab-based chemoimmunotherapy. J Cachexia Sarcopenia Muscle. (2017) 8:298–304. doi: 10.1002/jcsm.12161, PMID: 27897411 PMC5377388

[31] Go SI Park MJ Song HN Kim HG Kang MH Kang JH. A comparison of pectoralis versus lumbar skeletal muscle indices for defining sarcopenia in diffuse large B-cell lymphoma- two are better than one. Oncotarget. (2017) 8:47007–19. doi: 10.18632/oncotarget.16552, PMID: 28388585 PMC5564540

[32] Jabbour J Manana B Zahreddine A Saade C Charafeddine M Bazarbachi A. Sarcopenic obesity derived from PET/CT predicts mortality in lymphoma patients undergoing hematopoietic stem cell transplantation. Curr Res Transl Med. (2019) 67:93–9. doi: 10.1016/j.retram.2018.12.001, PMID: 30583985

[33] DeFilipp Z Troschel FM Qualls DA Li S Kuklinski MW Kempner ME. Evolution of body composition follow- ing autologous and allogeneic hematopoietic cell transplantation: incidence of sarcopenia and association with clinical outcomes. Biol Blood Marrow Transplant. (2018) 24:1741–7. doi: 10.1016/j.bbmt.2018.02.016 29496561

[34] Burkart M Schieber M Basu S Shah P Venugopal P Borgia JA. Evaluation of the impact of cachexia on clinical outcomes in aggressive lymphoma. Br J Haematol. (2019) 186:45–53. doi: 10.1111/bjh.15889, PMID: 30941741

[35] Go SI Kim HG Kang MH Park S Lee GW. Prognostic model based on the geriatric nutritional risk index and sarcopenia in patients with diffuse large B-cell lymphoma. BMC Cancer. (2020) 20:439. doi: 10.1186/s12885-020-06921-2, PMID: 32423395 PMC7236094

[36] Lin RJ Michaud L Lobaugh SM Mauguen A Elko TA Ruiz JD. The geriatric syndrome of sarcopenia impacts allogeneic hematopoietic cell transplantation outcomes in older lymphoma patients. Leuk Lymphoma. (2020) 61:1833–41. doi: 10.1080/10428194.2020.1742909, PMID: 32228298 PMC7429343

[37] Mishra A Bigam KD Extermann M Faramand R Thomas K Pidala JA. Sarcopenia and low muscle radiodensity associate with impaired FEV 1 in allogeneic haematopoietic stem cell transplant recipients. J Cachexia Sarcopenia Muscle. (2020) 11:1570–9. doi: 10.1002/jcsm.12604, PMID: 32729255 PMC7749567

[38] Rier HN Kharagjitsing H van Rosmalen J van Vugt J Westerweel PE de Jongh E. Prognostic impact of low muscle mass and low muscle density in patients with diffuse large B-cell lymphoma. Leuk Lymphoma. (2020) 61:1618–26. doi: 10.1080/10428194.2020.1737686, PMID: 32167390

[39] Armenian SH Iukuridze A The JB Mascarenhas K Herrera A McCune JS. Abnormal body composition is a predictor of adverse outcomes after autologous haematopoietic cell transplantation. J Cachexia Sarcopenia Muscle. (2020) 11:962–72. doi: 10.1002/jcsm.12570, PMID: 32212263 PMC7432567

[40] Bas V Umit EG Korkmaz U Baysal M Karaman Gulsaran S. Sarcopenia in Hodgkin’s lymphoma evaluated with 18-FDG PET/CT, focus on age, performance, and treatment. Support Care Cancer. (2021) 29:2475–80. doi: 10.1007/s00520-020-05772-8 32929535

[41] Lucijanić M Huzjan Korunić R Ivić M Fazlić Džankić A Jonjić Ž Mitrović Z. Psoas muscle index at the time of diagnosis might reflect the prognosis of classical Hodgkin’s lymphoma patients. Wien Klin Wochenschr. (2021) 9:1–3. doi: 10.1007/s00508-021-01850-x 33835267

[42] Hirota K Matsuse H Koya S Hashida R Bekki M Yanaga Y. Risks of muscle atrophy in patients with malignant lymphoma after autologous stem cell transplantation. Phys Ther Res. (2020) 24:69–76. doi: 10.1298/ptr.E10041 33981529 PMC8111411

[43] Guo J Cai P Li P Cao C Zhou J Dong L. Body composition as a predictor of toxicity and prognosis in patients with diffuse large B-cell lymphoma receiving R-CHOP immunochemotherapy. Curr Oncol. (2021) 28:1325–37. doi: 10.3390/curroncol28020126 33806839 PMC8025815

[44] Iltar U Sözel H Sözel YK Ataş Ü Yücel OK Salim O. Prognostic impact of the psoas muscle index, a parameter of sarcopenia, in patients with diffuse large B-cell lymphoma treated with rituximab-based chemoimmunotherapy. Leuk Lymphoma. (2021) 62:1098–106. doi: 10.1080/10428194.2020.1856833 33300380

[45] Besutti G Massaro F Bonelli E Braglia L Casali M Versari A. Prognostic impact of muscle quantity and quality and fat distribution in diffuse large B-cell lymphoma patients. Front Nutr. (2021) 8:620696. doi: 10.3389/fnut.2021.620696, PMID: 34026803 PMC8138563

[46] Zilioli VR Albano D Arcari A Merli F Coppola A Besutti G. Clinical and prognostic role of sarcopenia in elderly patients with classical Hodgkin lymphoma: a multicentre experience. J Cachexia Sarcopenia Muscle. (2021) 12:1042–55. doi: 10.1002/jcsm.12736, PMID: 34114749 PMC8350211

[47] Albano D Pasinetti N Dondi F Giubbini R Tucci A Bertagna F. Prognostic role of pre-treatment metabolic parameters and sarcopenia derived by 2-[^18^F]-FDG PET/CT in elderly mantle cell lymphoma. J Clin Med. (2022) 11:1210. doi: 10.3390/jcm11051210, PMID: 35268301 PMC8911178

[48] Albano D Dondi F Treglia G Tucci A Ravanelli M Farina D. Longitudinal body composition changes detected by [^18^F]FDG PET/CT during and after chemotherapy and their prognostic role in elderly Hodgkin lymphoma. Cancers. (2022) 14:5147. doi: 10.3390/cancers14205147, PMID: 36291931 PMC9601090

[49] Tan X Yuan H Li D Sun X Ding C Jiang L. Clinical and prognostic role of 2-[^18^F]FDG PET/CT and sarcopenia in treatment-naïve patients with T-cell lymphoblastic lymphoma. Ann Hematol. (2022) 101:2699–709. doi: 10.1007/s00277-022-04988-4 36123452

[50] Pénichoux J Lanic H Thill C Ménard AL Camus V Stamatoullas A. Prognostic relevance of sarcopenia, geriatric, and nutritional assessments in older patients with diffuse large B-cell lymphoma: results of a multicentric prospective cohort study. Ann Hematol. (2023) 102:1811–23. doi: 10.1007/s00277-023-05200-x, PMID: 37058153 PMC10260702

[51] Go SI Choi BH Park MJ Park S Kang MH Kim HG. Prognostic impact of pretreatment skeletal muscle index and CONUT score in diffuse large B-cell lymphoma. BMC Cancer. (2023) 23:1071. doi: 10.1186/s12885-023-11590-y, PMID: 37932700 PMC10629181

[52] Liao PH Chuang YH Yang FS Kuo CY Ma MC. Combination of sarcopenia and Anemia predicts worse outcome in elderly patients with diffuse large B-cell lymphoma. In Vivo. (2023) 37:1847–56. doi: 10.21873/invivo.13276, PMID: 37369473 PMC10347930

[53] Aleixo GFP Wei W Chen PH Gandhi NS Anwer F Dean R. The association of body composition and outcomes following autologous hematopoietic stem cell transplantation in patients with non-Hodgkin lymphoma. Bone Marrow Transplant. (2023) 58:1384–9. doi: 10.1038/s41409-023-02104-2, PMID: 37699993

[54] Chen Y Chen Z Tan X Zhang Q Zhou Y Yuan H. Role of body composition and metabolic parameters extracted from baseline ^18^F-FDG PET/CT in patients with diffuse large B-cell lymphoma. Ann Hematol. (2023) 102:2779–89. doi: 10.1007/s00277-023-05379-z 37530853

[55] Rejeski K Cordas dos Santos DM Parker NH Bücklein VL Winkelmann M Jhaveri KS. Influence of adipose tissue distribution, sarcopenia, and nutritional status on clinical outcomes after CD19 CAR T-cell therapy. Cancer Immunol Res. (2023) 11:707–19. doi: 10.1158/2326-6066.CIR-22-0487, PMID: 37040425 PMC10236147

[56] Sumransub N Cao Q Juckett M Betts B Holtan S Jurdi NE. Sarcopenia predicts inferior progression-free survival in lymphoma patients treated with autologous hematopoietic stem cell transplantation. Transplant Cell Ther. (2023) 29:263.e1–7. doi: 10.1016/j.jtct.2023.01.015 36682471

[57] Tan X Sun X Chen Y Wang F Shang Y Zhang Q. Implications of sarcopenia and Glucometabolism parameters of muscle derived from baseline and end-of-treatment ^18^F-FDG PET/CT in diffuse large B-cell lymphoma. Korean J Radiol. (2024) 25:277–88. doi: 10.3348/kjr.2023.0949, PMID: 38413112 PMC10912500

[58] Surov A Meyer HJ Hinnerichs M Ferraro V Zeremski V Mougiakakos D. CT-defined sarcopenia predicts treatment response in primary central nervous system lymphomas. Eur Radiol. (2024) 34:790–6. doi: 10.1007/s00330-023-09712-y, PMID: 37178198

[59] Niiyama-Uchibori Y Okamoto H Miyashita A Mizuhara K Kanayama-Kawaji Y Fujino T. Skeletal muscle index impacts the treatment outcome of elderly patients with diffuse large B cell lymphoma. Hematol Oncol. (2024) 42:e3252. doi: 10.1002/hon.3252 38287527

[60] Albano D Camoni L Rinaldi R Tucci A Zilioli VR Muzi C. Comparison between skeletal muscle and adipose tissue measurements with high-dose CT and low-dose attenuation correction CT of 18F-FDG PET/CT in elderly Hodgkin lymphoma patients: a two-Centre validation. Br J Radiol. (2021) 94:20200672. doi: 10.1259/bjr.20200672, PMID: 34106736 PMC8248212

[61] Furtner J Nenning KH Roetzer T Gesperger J Seebrecht L Weber M. Evaluation of the temporal muscle thickness as an independent prognostic biomarker in patients with primary central nervous system lymphoma. Cancers. (2021) 13:566. doi: 10.3390/cancers13030566, PMID: 33540564 PMC7867149

[62] Leone R Sferruzza G Calimeri T Steffanoni S Conte GM De Cobelli F. Quantitative muscle mass biomarkers are independent prognosis factors in primary central nervous system lymphoma: the role of L3-skeletal muscle index and temporal muscle thickness. Eur J Radiol. (2021) 143:109945. doi: 10.1016/j.ejrad.2021.109945, PMID: 34492625

[63] Xu T Li Y Liu Y Ning B Wu H Wei Y. Clinical and prognostic role of sarcopenia based on masticatory muscle index on MR images in patients with extranodal natural killer/T cell lymphoma, nasal type. Ann Hematol. (2023) 102:3521–32. doi: 10.1007/s00277-023-05436-7, PMID: 37702822

[64] Kim CY Hong CM Kim DH Son SH Jeong SY Lee SW. Prognostic value of whole-body metabolic tumour volume and total lesion glycolysis measured on 18F-FDG PET/CT in patients with extranodal NK/T-cell lymphoma. Eur J Nucl Med Mol Imaging. (2013) 40:1321–9. doi: 10.1007/s00259-013-2443-6, PMID: 23674211

[65] Kanoun S Rossi C Berriolo-Riedinger A Dygai-Cochet I Cochet A Humbert O. Baseline metabolic tumour volume is an independent prognostic factor in Hodgkin lymphoma. Eur J Nucl Med Mol Imaging. (2014) 41:1735–43. doi: 10.1007/s00259-014-2783-x 24811577

[66] Ceriani L Martelli M Zinzani PL Ferreri AJM Botto B Stelitano C. Utility of baseline 18FDG-PET/CT functional parameters in defining prognosis of primary mediastinal (thymic) large B-cell lymphoma. Blood. (2015) 126:950–6. doi: 10.1182/blood-2014-12-616474, PMID: 26089397

[67] Ilyas H Mikhaeel NG Dunn JT Rahman F Møller H Smith D. Defining the optimal method for measuring baseline metabolic tumour volume in diffuse large B cell lymphoma. Eur J Nucl Med Mol Imaging. (2018) 45:1142–54. doi: 10.1007/s00259-018-3953-z, PMID: 29460024 PMC5953976

[68] Royston P Altman DG Sauerbrei W. Dichotomizing continuous predictors in multiple regression: a bad idea. Stat Med. (2006) 25:127–41. doi: 10.1002/sim.2331, PMID: 16217841

[69] Boellaard R Buvat I Nioche C Ceriani L Cottereau A-S Guerra L. International benchmark for Total metabolic tumor volume measurement in baseline 18F-FDG PET/CT of lymphoma patients: a milestone toward clinical implementation. J Nucl Med. (2024) 65:1343–8. doi: 10.2967/jnumed.124.267789, PMID: 39089812 PMC11372260

[70] Guo B Tan X Ke Q Cen H. Prognostic value of baseline metabolic tumor volume and total lesion glycolysis in patients with lymphoma: a meta-analysis. PLoS One. (2019) 14:e0210224. doi: 10.1371/journal.pone.0210224, PMID: 30625203 PMC6326501

[71] Vercellino L Cottereau A-S Casasnovas O Tilly H Feugier P Chartier L. High total metabolic tumor volume at baseline predicts survival independent of response to therapy. Blood. (2020) 135:1396–405. doi: 10.1182/blood.2019003526, PMID: 31978225 PMC7162688

[72] Mikhaeel NG Heymans MW Eertink JJ De Vet HCW Boellaard R Dührsen U. Proposed new dynamic prognostic index for diffuse large B-cell lymphoma: international metabolic prognostic index. J Clin Oncol. (2022) 40:2352–60. doi: 10.1200/JCO.21.02063, PMID: 35357901 PMC9287279

[73] Winkelmann M Blumenberg V Rejeski K Bücklein VL Ruzicka M Unterrainer M. Prognostic value of the international metabolic prognostic index for lymphoma patients receiving chimeric antigen receptor T-cell therapy. Eur J Nucl Med Mol Imaging. (2023) 50:1406–13. doi: 10.1007/s00259-022-06075-2, PMID: 36513818

[74] Alderuccio JP Reis IM Hamadani M Nachiappan M Leslom S Kahl BS. PET/CT biomarkers enable risk stratification of patients with relapsed/refractory diffuse large B-cell lymphoma enrolled in the LOTIS-2 clinical trial. Clin Cancer Res Off J Am Assoc Cancer Res. (2024) 30:139–49. doi: 10.1158/1078-0432.CCR-23-1561, PMID: 37855688 PMC10872617

[75] Michaud L Bantilan K Mauguen A Moskowitz CH Zelenetz AD Schöder H. Prognostic value of 18F-FDG PET/CT in diffuse large B-cell lymphoma treated with a risk-adapted Immunochemotherapy regimen. J Nucl Med Off Publ Soc Nucl Med. (2023) 64:536–41. doi: 10.2967/jnumed.122.264740, PMID: 36549918 PMC10071786

[76] Duffles G da Silva Maués JH Lupinacci F Pereira LG Ferreira EN Freitas L. Circulating tumor DNA in diffuse large B-cell lymphoma: analysis of response assessment, correlation with PET/CT and clone evolution. Hematol Transfus Cell Ther. (2024) S2531-1379:00326–2. doi: 10.1016/j.htct.2024.07.005, PMID: 39317576 PMC11726095

[77] Ardeshna KM Qian W Smith P Braganca N Lowry L Patrick P. Rituximab versus a watch-and-wait approach in patients with advanced-stage, asymptomatic, non-bulky follicular lymphoma: an open-label randomised phase 3 trial. Lancet Oncol. (2014) 15:424–35. doi: 10.1016/S1470-2045(14)70027-0, PMID: 24602760

[78] Brice P Bastion Y Lepage E Brousse N Haïoun C Moreau P. Comparison in low-tumor-burden follicular lymphomas between an initial no-treatment policy, prednimustine, or interferon alfa: a randomized study from the Groupe d’Etude des Lymphomes Folliculaires. J Clin Oncol. (1997) 15:1110–7. doi: 10.1200/JCO.1997.15.3.1110, PMID: 9060552

[79] Zelenetz AD Gordon LI Chang JE Christian B Abramson JS Advani RH. NCCN guidelines® insights: B-cell lymphomas, version 5.2021: featured updates to the NCCN guidelines. J Natl Compr Cancer Netw. (2021) 19:1218–30. doi: 10.6004/jnccn.2021.0054, PMID: 34781267

[80] Yang Q Zhang H Zhang Y Zhang W Zhou D Luo Y. Baseline 18F-FDG PET/CT may contribute to the determination of initial treatment strategy for newly diagnosed follicular lymphoma. Eur J Radiol. (2024) 178:111632. doi: 10.1016/j.ejrad.2024.111632 39059082

[81] Cottereau AS Versari A Luminari S Dupuis J Chartier L Casasnovas R-O. Prognostic model for high-tumor-burden follicular lymphoma integrating baseline and end-induction PET: a LYSA/FIL study. Blood. (2018) 131:2449–53. doi: 10.1182/blood-2017-11-816298 29559480

[82] Zhou Y Zhao Z Li J Zhang B Sang S Wu Y. Prognostic values of baseline, interim and end-of therapy 18F-FDG PET/CT in patients with follicular lymphoma. Cancer Manag Res. (2019) 11:6871–85. doi: 10.2147/CMAR.S216445, PMID: 31413633 PMC6662523

[83] Liang J-H Zhang Y-P Xia J Ding C-Y Wu W Wang L. Prognostic value of baseline and interim Total metabolic tumor volume and Total lesion glycolysis measured on 18F-FDG PET-CT in patients with follicular lymphoma. Cancer Res Treat. (2019) 51:1479–87. doi: 10.4143/crt.2018.649 30913868 PMC6790864

[84] Song M-K Chung J-S Lee J-J Jeong SY Lee S-M Hong J-S. Metabolic tumor volume by positron emission tomography/computed tomography as a clinical parameter to determine therapeutic modality for early stage Hodgkin’s lymphoma. Cancer Sci. (2013) 104:1656–61. doi: 10.1111/cas.12282, PMID: 24033666 PMC7653520

[85] Tseng D Rachakonda LP Su Z Advani R Horning S Hoppe RT. Interim-treatment quantitative PET parameters predict progression and death among patients with Hodgkin’s disease. Radiat Oncol. (2012) 7:5. doi: 10.1186/1748-717X-7-5, PMID: 22260710 PMC3398283

[86] Yadgarov MY Dunaykin MM Shestopalov GI Kailash C Kireeva ED Myakova NV. Prognostic value of baseline and interim [18F]FDG PET metabolic parameters in pediatric Hodgkin’s lymphoma. Eur J Nucl Med Mol Imaging. (2024) 51:1955–64. doi: 10.1007/s00259-024-06643-8, PMID: 38351389

[87] Al-Ibraheem A Abdlkadir AS Al-Adhami DA Sathekge M Bom HH Ma'koseh M. The prognostic utility of 18F-FDG PET parameters in lymphoma patients under CAR-T-cell therapy: a systematic review and meta-analysis. Front Immunol. (2024) 15:1424269. doi: 10.3389/fimmu.2024.1424269, PMID: 39286245 PMC11402741

[88] Nioche C Orlhac F Boughdad S Reuzé S Goya-Outi J Robert C. LIFEx: A freeware for Radiomic feature calculation in multimodality imaging to accelerate advances in the characterization of tumor heterogeneity. Cancer Res. (2018) 78:4786–9. doi: 10.1158/0008-5472.CAN-18-0125, PMID: 29959149

[89] Cottereau AS Meignan M Nioche C Clerc J Chartier L Vercellino L. New approaches in characterization of lesions dissemination in DLBCL patients on baseline PET/CT. Cancers. (2021) 13:3998. doi: 10.3390/cancers13163998, PMID: 34439152 PMC8392801

[90] Cottereau AS Nioche C Dirand AS Clerc J Morschhauser F Casasnovas O. ^18^F-FDG PET dissemination features in diffuse large B-cell lymphoma are predictive of outcome. J Nucl Med. (2020) 61:40–5. doi: 10.2967/jnumed.119.229450, PMID: 31201248 PMC6954460

[91] Cottereau AS Meignan M Nioche C Capobianco N Clerc J Chartier L. Risk stratification in diffuse large B-cell lymphoma using lesion dissemination and metabolic tumor burden calculated from baseline PET/CT^†^. Ann Oncol. (2021) 32:404–11. doi: 10.1016/j.annonc.2020.11.019, PMID: 33278600

[92] Girum KB Rebaud L Cottereau AS Meignan M Clerc J Vercellino L. ^18^F-FDG PET maximum-intensity projections and artificial intelligence: A win-win combination to easily measure prognostic biomarkers in DLBCL patients. J Nucl Med. (2022) 63:1925–32. doi: 10.2967/jnumed.121.263501, PMID: 35710733 PMC9730929

[93] Xu H Ma J Yang G Xiao S Li W Sun Y. Prognostic value of metabolic tumor volume and lesion dissemination from baseline PET/CT in patients with diffuse large B-cell lymphoma: further risk stratification of the group with low-risk and high-risk NCCN-IPI. Eur J Radiol. (2023) 163:110798. doi: 10.1016/j.ejrad.2023.110798, PMID: 37030099

[94] Eertink JJ van de Brug T Wiegers SE Zwezerijnen GJC Pfaehler EAG Pj L. ^18^F-FDG PET baseline radiomics features improve the prediction of treatment outcome in diffuse large B-cell lymphoma. Eur J Nucl Med Mol Imaging. (2022) 49:932–42. doi: 10.1007/s00259-021-05480-3, PMID: 34405277 PMC8803694

[95] Eertink JJ Zwezerijnen GJC Cysouw MCF Wiegers SE Pfaehler EAG Lugtenburg PJ. Comparing lesion and feature selections to predict progression in newly diagnosed DLBCL patients with FDG PET/CT radiomics features. Eur J Nucl Med Mol Imaging. (2022) 49:4642–51. doi: 10.1007/s00259-022-05916-4, PMID: 35925442 PMC9606052

[96] Ceriani L Milan L Cascione L Gritti G Dalmasso F Esposito F. Generation and validation of a PET radiomics model that predicts survival in diffuse large B cell lymphoma treated with R-CHOP14: A SAKK 38/07 trial post-hoc analysis. Hematol Oncol. (2022) 40:11–21. doi: 10.1002/hon.2935, PMID: 34714558

[97] Dang J Peng X Wu P Yao Y Tan X Ye Z. Predictive value of Dmax and %ΔSUVmax of 18F-FDG PET/CT for the prognosis of patients with diffuse large B-cell lymphoma. BMC Med Imaging. (2023) 23:173. doi: 10.1186/s12880-023-01138-8, PMID: 37907837 PMC10617085

[98] Jo JH Chung HW Kim SY Lee MH So Y. FDG PET/CT maximum tumor dissemination to predict recurrence in patients with diffuse large B-cell lymphoma. Nucl Med Mol Imaging. (2023) 57:26–33. doi: 10.1007/s13139-022-00782-2, PMID: 36643943 PMC9832207

[99] Marchal E Palard-Novello X Lhomme F Meyer ME Manson G Devillers A. Baseline [^18^F]FDG PET features are associated with survival and toxicity in patients treated with CAR T cells for large B cell lymphoma. Eur J Nucl Med Mol Imaging. (2024) 51:481–9. doi: 10.1007/s00259-023-06427-6 37721580

[100] Weisman AJ Kim J Lee I McCarten KM Kessel S Schwartz CL. Automated quantification of baseline imaging PET metrics on FDG PET/CT images of pediatric Hodgkin lymphoma patients. EJNMMI Phys. (2020) 7:76. doi: 10.1186/s40658-020-00346-3, PMID: 33315178 PMC7736382

[101] Driessen J Zwezerijnen GJC Schöder H Drees EEE Kersten MJ Moskowitz AJ. The impact of semiautomatic segmentation methods on metabolic tumor volume, intensity, and dissemination Radiomics in ^18^F-FDG PET scans of patients with classical Hodgkin lymphoma. J Nucl Med. (2022) 63:1424–30. doi: 10.2967/jnumed.121.263067, PMID: 34992152 PMC9454468

[102] Zhou Y Zhu Y Chen Z Li J Sang S Deng S. Radiomic features of ^18^F-FDG PET in Hodgkin lymphoma are predictive of outcomes. Contrast Media Mol Imaging. (2021) 2021:6347404. doi: 10.1155/2021/6347404, PMID: 34887712 PMC8629643

[103] Durmo R Donati B Rebaud L Cottereau AS Ruffini A Nizzoli ME. Prognostic value of lesion dissemination in doxorubicin, bleomycin, vinblastine, and dacarbazine-treated, interimPET-negative classical Hodgkin lymphoma patients: a radio-genomic study. Hematol Oncol. (2022) 40:645–57. doi: 10.1002/hon.3025, PMID: 35606338 PMC9796042

[104] Drees EEE Driessen J Zwezerijnen GJC Verkuijlen SAWM Eertink JJ van Eijndhoven MAJ. Blood-circulating EV-miRNAs, serum TARC, and quantitative FDG-PET features in classical Hodgkin lymphoma. EJHaem. (2022) 3:908–12. doi: 10.1002/jha2.432 36051072 PMC9422001

[105] Li H Wang M Zhang Y Hu F Wang K Wang C. Prediction of prognosis and pathologic grade in follicular lymphoma using ^18^F-FDG PET/CT. Front Oncol. (2022) 12:943151. doi: 10.3389/fonc.2022.943151, PMID: 35965552 PMC9366037

[106] Xie Y Teng Y Jiang C Ding C Zhou Z. Prognostic value of 18F-FDG lesion dissemination features in patients with peripheral T-cell lymphoma (PTCL). Jpn J Radiol. (2023) 41:777–86. doi: 10.1007/s11604-023-01398-y 36752954

[107] Albano D Calabrò A Talin A Dondi F Pagani C Tucci A. 2-[^18^]F FDG PET/CT dissemination features in adult burkitt lymphoma are predictive of outcome. Ann Hematol. (2024) 103:2419–27. doi: 10.1007/s00277-024-05672-5 38374254

[108] Gong H Tang B Li T Li J Tang L Ding C. The added prognostic values of baseline PET dissemination parameter in patients with angioimmunoblastic T-cell lymphoma. EJHaem. (2022) 4:67–77. doi: 10.1002/jha2.610, PMID: 36819177 PMC9928789

[109] Vergote VKJ Verhoef G Janssens A Woei-A-Jin FJSH Laenen A Tousseyn T. [^18^F]FDG-PET/CT volumetric parameters can predict outcome in untreated mantle cell lymphoma. Leuk Lymphoma. (2023) 64:161–70. doi: 10.1080/10428194.2022.2131415, PMID: 36223113

[110] Frood R Burton C Tsoumpas C Frangi AF Gleeson F Patel C. Baseline PET/CT imaging parameters for prediction of treatment outcome in Hodgkin and diffuse large B cell lymphoma: a systematic review. Eur J Nucl Med Mol Imaging. (2021) 48:3198–220. doi: 10.1007/s00259-021-05233-2, PMID: 33604689 PMC8426243

[111] Rizzo A Triumbari EKA Gatta R Boldrini L Racca M Mayerhoefer M. The role of 18F-FDG PET/CT radiomics in lymphoma. Clin Translat Imaging. (2021) 9:589–98. doi: 10.1007/s40336-021-00451-y

[112] Ko KY Liu CJ Ko CL Yen RF. Intratumoral heterogeneity of pretreatment 18F-FDG PET images predict disease progression in patients with nasal type Extranodal natural killer/T-cell lymphoma. Clin Nucl Med. (2016) 41:922–6. doi: 10.1097/RLU.0000000000001375, PMID: 27749404

[113] Ben Bouallègue F Tabaa YA Kafrouni M Cartron G Vauchot F Mariano-Goulart D. Association between textural and morphological tumor indices on baseline PET-CT and early metabolic response on interim PET-CT in bulky malignant lymphomas. Med Phys. (2017) 44:4608–19. doi: 10.1002/mp.12349 28513853

[114] Parvez A Tau N Hussey D Maganti M Metser U. 18F-FDG PET/CT metabolic tumor parameters and radiomics features in aggressive non-Hodgkin's lymphoma as predictors of treatment outcome and survival. Ann Nucl Med. (2018) 32:410–6. doi: 10.1007/s12149-018-1260-1 29754276

[115] Lue KH Wu YF Liu SH Hsieh TC Chuang KS Lin HH. Intratumor heterogeneity assessed by 18F-FDG PET/CT predicts treatment response and survival outcomes in patients with Hodgkin lymphoma. Acad Radiol. (2020) 27:e183–92. doi: 10.1016/j.acra.2019.10.015, PMID: 31761665

[116] Lue KH Wu YF Liu SH Hsieh TC Chuang KS Lin HH. Prognostic value of pretreatment Radiomic features of 18F-FDG PET in patients with Hodgkin lymphoma. Clin Nucl Med. (2019) 44:e559–65. doi: 10.1097/RLU.0000000000002732, PMID: 31306204

[117] Mayerhoefer ME Riedl CC Kumar A Gibbs P Weber M Tal I. Radiomic features of glucose metabolism enable prediction of outcome in mantle cell lymphoma. Eur J Nucl Med Mol Imaging. (2019) 46:2760–9. doi: 10.1007/s00259-019-04420-6 31286200 PMC6879438

[118] Milgrom SA Elhalawani H Lee J Wang Q Mohamed ASR Dabaja BS. A PET Radiomics model to predict refractory mediastinal Hodgkin lymphoma. Sci Rep. (2019) 9:1322. doi: 10.1038/s41598-018-37197-z, PMID: 30718585 PMC6361903

[119] Tatsumi M Isohashi K Matsunaga K Watabe T Kato H Kanakura Y. Volumetric and texture analysis on FDG PET in evaluating and predicting treatment response and recurrence after chemotherapy in follicular lymphoma. Int J Clin Oncol. (2019) 24:1292–300. doi: 10.1007/s10147-019-01482-2, PMID: 31165310

[120] Wang H Zhao S Li L Tian R. Development and validation of an 18F-FDG PET radiomic model for prognosis prediction in patients with nasal-type extranodal natural killer/T cell lymphoma. Eur Radiol. (2020) 30, 10:5578–87. doi: 10.1007/s00330-020-06943-1, PMID: 32435928

[121] Wu J Lian C Ruan S Mazur TR Mutic S Anastasio MA. Treatment outcome prediction for Cancer patients based on Radiomics and belief function theory. IEEE Trans Radiat Plasma Med Sci. (2019) 3:216–24. doi: 10.1109/TRPMS.2018.2872406, PMID: 31903444 PMC6941853

[122] Zhou Y Ma XL Pu LT Zhou RF Ou XJ Tian R. Prediction of overall survival and progression-free survival by the 18F-FDG PET/CT Radiomic features in patients with primary gastric diffuse large B-cell lymphoma. Contrast Media Mol Imaging. (2019) 2019:5963607. doi: 10.1155/2019/5963607, PMID: 31777473 PMC6875372

[123] Aide N Fruchart C Nganoa C Gac AC Lasnon C. Baseline 18F-FDG PET radiomic features as predictors of 2-year event-free survival in diffuse large B cell lymphomas treated with immunochemotherapy. Eur Radiol. (2020) 30:4623–32. doi: 10.1007/s00330-020-06815-8, PMID: 32248365

[124] Rodríguez Taroco MG Cuña EG Pages C Schelotto M González-Sprinberg GA Castillo LA. Prognostic value of imaging markers from 18FDG-PET/CT in paediatric patients with Hodgkin lymphoma. Nucl Med Commun. (2021) 42:306–14. doi: 10.1097/MNM.0000000000001337, PMID: 33306628

[125] Sun Y Qiao X Jiang C Liu S Zhou Z. Texture analysis improves the value of pretreatment 18F-FDG PET/CT in predicting interim response of primary gastrointestinal diffuse large B-cell lymphoma. Contrast Media Mol Imaging. (2020) 2020:2981585. doi: 10.1155/2020/2981585, PMID: 32922221 PMC7463417

[126] Wang M Xu H Xiao L Song W Zhu S Ma X. Prognostic value of functional parameters of 18F-FDG-PET images in patients with primary renal/adrenal lymphoma. Contrast Media Mol Imaging. (2019) 2019:2641627. doi: 10.1155/2019/2641627, PMID: 31427906 PMC6683818

[127] Lue KH Wu YF Lin HH Hsieh TC Liu SH Chan SC. Prognostic value of baseline radiomic features of 18F-FDG PET in patients with diffuse large B-cell lymphoma. Diagnostics. (2020) 11:36. doi: 10.3390/diagnostics11010036, PMID: 33379166 PMC7824203

[128] Lartizien C Rogez M Niaf E Ricard F. Computer-aided staging of lymphoma patients with FDG PET/CT imaging based on textural information. IEEE J Biomed Health Inform. (2014) 18:946–55. doi: 10.1109/JBHI.2013.2283658, PMID: 24081876

[129] Kong Z Jiang C Zhu R Feng S Wang Y Li J. 18F-FDG-PET-based radiomics features to distinguish primary central nervous system lymphoma from glioblastoma. Neuroimage Clin. (2019) 23:101912. doi: 10.1016/j.nicl.2019.101912 31491820 PMC6702330

[130] Lippi M Gianotti S Fama A Casali M Barbolini E Ferrari A. Texture analysis and multiple-instance learning for the classification of malignant lymphomas. Comput Methods Prog Biomed. (2020) 185:105153. doi: 10.1016/j.cmpb.2019.105153, PMID: 31678792

[131] Ou X Wang J Zhou R Zhu S Pang F Zhou Y. Ability of 18F-FDG PET/CT Radiomic features to distinguish breast carcinoma from breast lymphoma. Contrast Media Mol Imaging. (2019) 2019:4507694. doi: 10.1155/2019/4507694, PMID: 30930700 PMC6410462

[132] Xu H Guo W Cui X Zhuo H Xiao Y Ou X. Three-dimensional texture analysis based on PET/CT images to distinguish hepatocellular carcinoma and hepatic lymphoma. Front Oncol. (2019) 9:844. doi: 10.3389/fonc.2019.00844, PMID: 31552173 PMC6733884

[133] Ou X Zhang J Wang J Pang F Wang Y Wei X. Radiomics based on 18 F-FDG PET/CT could differentiate breast carcinoma from breast lymphoma using machine-learning approach: a preliminary study. Cancer Med. (2020) 9:496–506. doi: 10.1002/cam4.2711, PMID: 31769230 PMC6970046

[134] Sun YW Ji CF Wang H He J Liu S Ge Y. Differentiating gastric cancer and gastric lymphoma using texture analysis (TA) of positron emission tomography (PET). Chin Med J. (2020) 134:439–47. doi: 10.1097/CM9.0000000000001206, PMID: 33230019 PMC7909296

[135] Aide N Talbot M Fruchart C Damaj G Lasnon C. Diagnostic and prognostic value of baseline FDG PET/CT skeletal textural features in diffuse large B cell lymphoma. Eur J Nucl Med Mol Imaging. (2018) 45:699–711. doi: 10.1007/s00259-017-3899-6, PMID: 29214417 PMC5978926

[136] Mayerhoefer ME Riedl CC Kumar A Dogan A Gibbs P Weber M. [18F]FDG-PET/CT Radiomics for prediction of bone marrow involvement in mantle cell lymphoma: a retrospective study in 97 patients. Cancers (Basel). (2020) 12:1138. doi: 10.3390/cancers12051138, PMID: 32370121 PMC7281173

[137] Kenawy MA Khalil MM Abdelgawad MH El-Bahnasawy HH. Correlation of texture feature analysis with bone marrow infiltration in initial staging of patients with lymphoma using 18F-fluorodeoxyglucose positron emission tomography combined with computed tomography. Pol J Radiol. (2020) 85:586–e594. doi: 10.5114/pjr.2020.99833, PMID: 33204373 PMC7654316

[138] Jimenez JE Dai D Xu G Zhao R Li T Pan T. Lesion-based Radiomics signature in Pretherapy 18F-FDG PET predicts treatment response to Ibrutinib in lymphoma. Clin Nucl Med. (2022) 47:209–18. doi: 10.1097/RLU.0000000000004060, PMID: 35020640 PMC8851692

[139] Frood R Clark M Burton C Tsoumpas C Frangi AF Gleeson F. Discovery of pre-treatment FDG PET/CT-derived Radiomics-based models for predicting outcome in diffuse large B-cell lymphoma. Cancers. (2022) 14:1711. doi: 10.3390/cancers14071711, PMID: 35406482 PMC8997127

[140] Jiang C Huang X Li A Teng Y Ding C Chen J. Radiomics signature from [18F]FDG PET images for prognosis predication of primary gastrointestinal diffuse large B cell lymphoma. Eur Radiol. (2022) 32:5730–41. doi: 10.1007/s00330-022-08668-9, PMID: 35298676

[141] Ortega C Eshet Y Prica A Anconina R Johnson S Constantini D. Combination of FDG PET/CT radiomics and clinical parameters for outcome prediction in patients with Hodgkin's lymphoma. Cancers. (2023) 15:2056. doi: 10.3390/cancers15072056, PMID: 37046717 PMC10093084

[142] Triumbari EKA Gatta R Maiolo E De Summa M Boldrini L Mayerhoefer ME. Baseline 18F-FDG PET/CT radiomics in classical Hodgkin's lymphoma: The predictive role of the largest and the hottest lesions. Diagnostics. (2023) 13:1391. doi: 10.3390/diagnostics13081391, PMID: 37189492 PMC10137254

[143] Li M Yao H Zhang P Zhang L Liu W Jiang Z. Development and validation of a [18F]FDG PET/CT-based radiomics nomogram to predict the prognostic risk of pretreatment diffuse large B cell lymphoma patients. Eur Radiol. (2023) 33:3354–65. doi: 10.1007/s00330-022-09301-5 36547676 PMC10121518

[144] Cui Y Jiang Y Deng X Long W Liu B Fan W. 18F-FDG PET-based combined baseline and end-of-treatment radiomics model improves the prognosis prediction in diffuse large B cell lymphoma after first-line therapy. Acad Radiol. (2023) 30:1408–18. doi: 10.1016/j.acra.2022.10.011 36437191

[145] Samimi R Shiri I Ahmadyar Y van den Hoff J Kamali-Asl A Rezaee AY. Radiomics predictive modeling from dual-time-point FDG PET Ki parametric maps: application to chemotherapy response in lymphoma. EJNMMI Res. (2023) 13:70. doi: 10.1186/s13550-023-01022-0, PMID: 37493872 PMC10371962

[146] Zhao S Wang J Jin C Zhang X Xue C Zhou R. Stacking ensemble learning-based [18F]FDG PET radiomics for outcome prediction in diffuse large B-cell lymphoma. J Nucl Med. (2023) 64:1603–9. doi: 10.2967/jnumed.122.265244, PMID: 37500261

[147] Jing F Liu Y Zhao X Wang N Dai M Chen X. Baseline 18F-FDG PET/CT radiomics for prognosis prediction in diffuse large B cell lymphoma. EJNMMI Res. (2023) 13:92. doi: 10.1186/s13550-023-01047-5, PMID: 37884763 PMC10603012

[148] Ligero M Simó M Carpio C Iacoboni G Balaguer-Montero M Navarro V. PET-based radiomics signature can predict durable responses to CAR T-cell therapy in patients with large B-cell lymphoma. EJHaem. (2023) 4:1081–8. doi: 10.1002/jha2.757, PMID: 38024636 PMC10660117

[149] Driessen J Zwezerijnen GJC Schöder H Kersten MJ Moskowitz AJ Moskowitz CH. Prognostic model using 18F-FDG PET radiomics predicts progression-free survival in relapsed/refractory Hodgkin lymphoma. Blood Adv. (2023) 7:6732–43. doi: 10.1182/bloodadvances.2023010404, PMID: 37722357 PMC10651466

[150] Carlier T Frécon G Mateus D Rizkallah M Kraeber-Bodéré F Kanoun S. Prognostic value of 18F-FDG PET Radiomics features at baseline in PET-guided consolidation strategy in diffuse large B-cell lymphoma: a machine-learning analysis from the GAINED study. J Nucl Med. (2024) 65:156–62. doi: 10.2967/jnumed.123.265872 37945379

[151] Luo Y Huang Z Gao Z Wang B Zhang Y Bai Y. Prognostic value of 18F-FDG PET/CT radiomics in extranodal nasal-type NK/T cell lymphoma. Korean J Radiol. (2024) 25:189–98. doi: 10.3348/kjr.2023.0618, PMID: 38288898 PMC10831304

[152] Albano D Calabrò A Dondi F Bagnasco S Tucci A Bertagna F. The role of baseline 2-[18 F]-FDG-PET/CT metrics and radiomics features in predicting primary gastric lymphoma diagnosis. Hematol Oncol. (2024) 42:e3266. doi: 10.1002/hon.3266 38444261

[153] Yousefirizi F Gowdy C Klyuzhin IS Sabouri M Tonseth P Hayden AR. Evaluating outcome prediction via baseline, end-of-treatment, and Delta Radiomics on PET-CT images of primary mediastinal large B-cell lymphoma. Cancers (Basel). (2024) 16:1090. doi: 10.3390/cancers16061090 38539425 PMC10968861

[154] Jing F Zhang X Liu Y Chen X Zhao J Zhao X. Baseline 18F-FDG PET/CT radiomics for prognosis prediction in diffuse large B cell lymphoma with extranodal involvement. Clin Transl Oncol. (2024) 31:1–9. doi: 10.1007/s12094-024-03633-y 39083140

[155] Jing F Zhang X Liu Y Chen X Zhao X Chen X. Baseline 18F-FDG PET Radiomics predicting therapeutic efficacy of diffuse large B-cell lymphoma after R-CHOP (-like) therapy. Cancer Biother Radiopharm. (2024). doi: 10.1089/cbr.2024.0115, PMID: 39230437

[156] Zhu S Xu H Shen C Wang Y Xu W Duan S. Differential diagnostic ability of 18F-FDG PET/CT radiomics features between renal cell carcinoma and renal lymphoma. Q J Nucl Med Mol Imaging. (2021) 65:72–8. doi: 10.23736/S1824-4785.19.03137-6, PMID: 31140234

[157] Lovinfosse P Ferreira M Withofs N Jadoul A Derwael C Frix AN. Distinction of lymphoma from sarcoidosis on 18F-FDG PET/CT: evaluation of Radiomics-feature-guided machine learning versus human reader performance. J Nucl Med. (2022) 63:1933–40. doi: 10.2967/jnumed.121.263598, PMID: 35589406 PMC9730930

[158] Han EJ O JH Yoon H Ha S Yoo IR Min JW. Comparison of FDG PET/CT and bone marrow biopsy results in patients with diffuse large B cell lymphoma with subgroup analysis of PET Radiomics. Diagnostics. (2022) 12:222. doi: 10.3390/diagnostics12010222, PMID: 35054389 PMC8774933

[159] Hasenclever D Kurch L Mauz-Körholz C Elsner A Georgi T Wallace H. qPET - a quantitative extension of the Deauville scale to assess response in interim FDG-PET scans in lymphoma. Eur J Nucl Med Mol Imaging. (2014) 41:1301–8. doi: 10.1007/s00259-014-2715-9 24604592

[160] Kumar A Jacob P Watts A Joseph A Kaur H Hooda M. Evaluation of reconstruction algorithms to validate the NEMA phantom results in clinical scenario - A comparative study using time-of-flight versus non-time-of-flight positron emission tomography imaging. Indian J Nucl Med. (2022) 37:113–20. doi: 10.4103/ijnm.ijnm_137_21, PMID: 35982821 PMC9380809

[161] Guralnik JM Simonsick EM Ferrucci L Glynn RJ Berkman LF Blazer DG. A short physical performance battery assessing lower extremity function: association with self-reported disability and prediction of mortality and nursing home admission. J Gerontol. (1994) 49:M85–94. doi: 10.1093/geronj/49.2.m85, PMID: 8126356

[162] Roberts HC Denison HJ Martin HJ Patel HP Syddall H Cooper C. A review of the measurement of grip strength in clinical and epidemiological studies: towards a standardised approach. Age Ageing. (2011) 40:423–9. doi: 10.1093/ageing/afr051 21624928

